# The Odor Delivery Optimization Research System (ODORS): An Open-Source Olfactometer for Behavioral Assessments in Tethered and Untethered Rodents

**DOI:** 10.1523/ENEURO.0161-25.2025

**Published:** 2025-12-17

**Authors:** Filip Kosel, Mackenzie R. Hartley, Tamara B. Franklin

**Affiliations:** Department of Psychology and Neuroscience, Dalhousie University, Halifax, Nova Scotia B3H 4R2, Canada

**Keywords:** olfaction, open behavior, rodent

## Abstract

Olfaction is the dominant sensory modality in rodents. It can be used to assess behavioral phenomena including stress, learning and memory, and social investigation, and impaired olfaction is implicated in several neurological disorders. Paradigms such as the olfactory habituation/dishabituation (OHD) task can assess olfactory perception, memory, and motivation. However, these tasks require manual stimulus presentation, introducing variability and making them labor-intensive. Olfactometers allow automated stimulus delivery, but the OHD task has not yet been adapted for use with an olfactometer. Additionally, current olfactometer designs require proprietary software or components that are difficult to obtain/fabricate and commercial units are expensive. As a result, these apparatuses have not been widely implemented. Here, we describe the design and assembly of the Odor Delivery Optimization Research System (ODORS), an economical, modular, and open-source olfactometer for use in rodents, and describe a variant of the OHD task that can be automated using this apparatus. The design is based on five principles: (1) familiar layout and function; (2) use of inexpensive, readily available components; (3) easily integrated, modular design; (4) real-time assessment of odorant levels; and (5) the ability to test tethered and untethered rodents in optogenetic and electrophysiological experiments. Male and female C57BL/6NCrl mice performing OHD in the ODORS exhibit the characteristic habituation to repeated presentations of an odor and dishabituation to the first presentation of a novel odor. As a result, we suggest that the ODORS makes improved olfactory testing accessible to many labs and offers a major refinement over existing OHD testing paradigms.

## Significance Statement

Olfaction is a key modality in animals, and evidence suggests some neurological disorders (e.g., Alzheimer's disease, Parkinson's disease, autism spectrum disorder) are linked with impaired olfaction. However, paradigms used to study olfaction in rodent models, including the olfactory habituation/dishabituation task, are subject to variability introduced by the experimenter and are labor-intensive to administer and score. Additionally, automated olfactometers are expensive and difficult to integrate with existing testing systems due to proprietary hardware and software. The apparatus and paradigm described herein provides an accessible alternative to existing systems and tasks, allowing labs of all sizes to contribute to this valuable field of research.

## Introduction

Rodent studies with olfactory stimuli have examined many behavioral phenomena, including stress responses (Wright et al., 2013), learning and memory (Roddick et al., 2014), and social investigation (Kosel et al., 2019), and the importance of odor cues in social behaviors is underscored by electrophysiological data (Levy et al., 2019). Several behavioral paradigms and apparatuses have been developed to standardize/facilitate testing with olfactory stimuli. Tasks based on habituation to repeated presentations of odor stimuli (Cleland et al., 2002; Yang and Crawley, 2009; Wesson et al., 2010) offer simple, low-cost methods for assessing olfactory perception, memory, and motivation. However, these tasks are time- and labor-intensive, and even with attempts to improve efficiency (Oummadi et al., 2020), available methods still require manual presentation of odor stimuli and introduce variability associated with the experimenter.

Olfactometers allow for automated and precise stimulus delivery, allowing administration of tasks such as the delayed matching-to-sample and olfactory sensitivity tasks (Roddick et al., 2014, 2016) and assessment of neural activity in response to specific stimuli (Levy et al., 2019). Currently, available olfactometers for animal testing fall into one of two categories: units which flood the chamber with odorized air using an inlet at one end of the testing chamber and an exhaust port at the other end (Levy et al., 2019) and units which use positive pressure to restrict odorized air behind an odor port (Bodyak and Slotnick, 1999; Roddick et al., 2014, 2016). However, commercially available units are expensive, and the use of proprietary hardware and software can preclude control of the apparatus directly from existing lab systems (e.g., behavioral, electrophysiological, or optogenetic software and equipment). While some previous publications describe the design and function of olfactometers (Fox and Thiessen, 1984; Bodyak and Slotnick, 1999; Slotnick and Bodyak, 2002; Rebello et al., 2015; Burton et al., 2019), these designs require components that may be difficult to obtain commercially or require advanced fabrication capabilities (e.g., borosilicate glass air manifolds, custom-made odorant vessels, or custom-machined odorant tube holders) or may be primarily designed for use with head-fixed animals (Rebello et al., 2015; Burton et al., 2019). Finally, tasks using stimulus odor concentrations based on simple dilution can lead to presentation of unknown odorant concentrations, and calculating final odorant concentrations using liquid- and vapor-phase concentrations of stimuli (Bodyak and Slotnick, 1999) can make it difficult to consistently test novel odorants where these values are unknown.

Here, we describe the design, assembly, and use of the Odor Delivery Optimization Research System (ODORS), a simplified, low-cost olfactometer for accurate and automated delivery of olfactory stimuli.

## Materials and Methods

**Figure 1.**
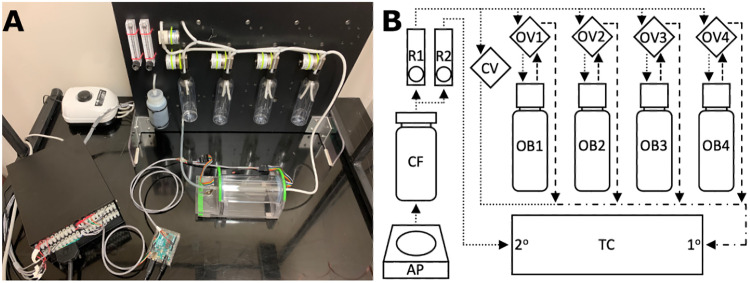
ODORS overview and air flow diagram. ***A***, Representative image of the ODORS, with the air pump (top left), odorant board (top right), testing chamber (middle right), Arduino (bottom center), and valve control box (bottom left). ***B***, Overall air flow diagram for the ODORS. Air from the air pump (AP) passes through the carbon filter (CF) and then splits into two rotameters (R1 and R2). Air from the primary rotameter (R1) supplies the single-tube clean air valve (CV) and the four double-tube odorant valves (OV1-4). Air through the clean air valve forms the beginning of the downstream feed to the testing chamber (TC) primary side (1°). Air passing through the odorant valves enters the respective odorant bottle (OB1–4) where it mixes with the odor stimulus, exits the bottle, passes back through the odorant valve, and joins the downstream feed to the testing chamber. Air from the secondary rotameter (R2) connects directly to the testing chamber secondary side (2°) to provide positive pressure to keep odorized air localized near the primary side of the chamber. Air from the testing chamber exits through the slot along the top of the testing chamber. Dotted lines indicate clean air lines, dashed lines indicate stimulus air lines, and the dashed/dotted line leading to the testing chamber primary side carries both clean and stimulus air. Arrows indicate direction of air flow.

The ODORS has three major components: the odorant board, testing chamber, and computer interface (Fig. 1). Our specific design and component choices will be discussed in detail, but components with similar specifications may provide suitable alternatives. The Bill of Materials is available at https://github.com/FilipKosel/ODORS.

### Licensing

The hardware outlined herein (including 3D models of end caps) is licensed under CC BY-NC-SA 4.0. To view a copy of this license, visit: https://creativecommons.org/licenses/by-nc-sa/4.0/.

The code outlined herein (available at https://github.com/FilipKosel/ODORS) is a free software; you can redistribute it and/or modify it under the terms of the GNU General Public License as published by the Free Software Foundation, either version 3 of the License or (at your option) any later version. To view a copy of this license, visit: https://www.gnu.org/licenses/.

### ODORS construction

Step-by-step images of the ODORS assembly process can be found as Extended Data.

#### Odorant board

**Figure 2.**
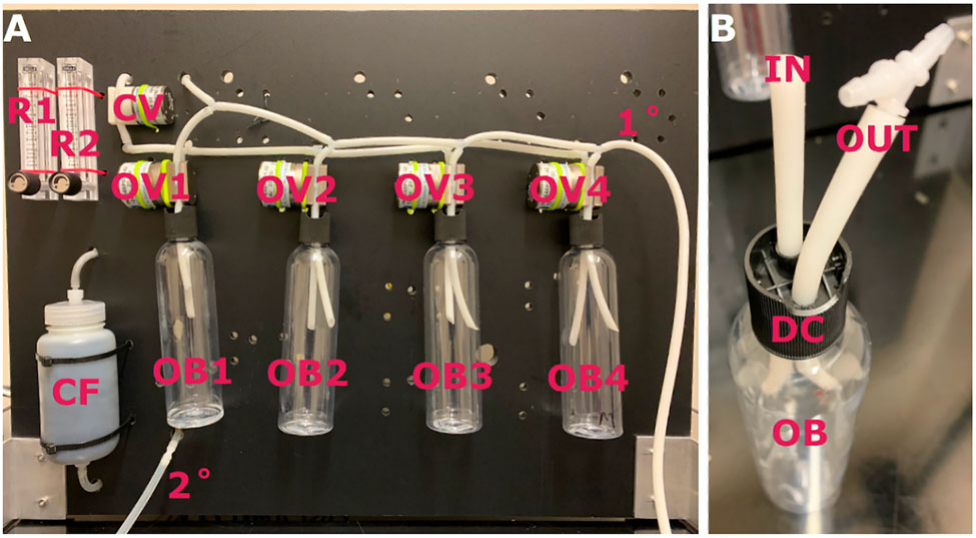
ODORS odorant board and bottle. ***A***, Representative image of the odorant board showing the location of the carbon filter (CF), rotameters (R1 and R2), clean air valves (CV), and odorized air valves (OV1–4). ***B***, Representative image of an odorant bottle (OB), including the modified 24-410 disc cap (DC). The lid from the cap has been removed, and the air line entering the bottle (IN) passes through the original 0.240″ (∼6.1 mm) hole visible in the back. A second 6 mm (∼15/64″) diameter hole was drilled on the opposite side of the cap (toward the front) for a second air line to exit the bottle (OUT); the barbed wye connector is visible on the end of the secondary line, allowing for the bottle and air lines to be easily replaced for use with different odorants.

*Function*: The odorant board consists of a 3′ × 2′ sheet of low-density polyethylene and provides a mounting surface for the air filter, rotameters, air valves, and stimulus bottles (Fig. 2), resulting in a lightweight, compact assembly that can be portable or mounted permanently.

*Air supply*: Air is supplied by an EcoPlus Eco Air 4 air pump (HGC728355; Hawthorne Gardening Company). C-Flex tubing (#06424-67; Cole-Parmer) and polyethylene super-flow barbed wye connectors (not PTFE-coated, 1/8″ tube; #2808K127; McMaster-Carr) were used to combine the outlets into a single air supply line.

*Air filter*: A custom-made air filter composed of a 500 ml Nalgene wide-mouth high–density polyethylene bottle (#2104-0016; Nalge Nunc International) drilled and tapped in the cap and on the bottom for barbed fittings (1/8″ tube by male 1/8″ NPT thread; #2808K22; McMaster-Carr) was used to reduce environmental odorants present in the air supply before it reached the odorant tubes. Activated carbon is commonly used as a filter medium to remove environmental pollutants from air or water (e.g., respirators, water filters) and is typically available in bulk form. As the material can settle due to handling or vibrations from the air pumps, the varying particle sizes present in granular carbon could settle and impede airflow over time. To reduce the likelihood of this occurring, we used uniformly sized spherical carbon beads (Matrix Carbon; Seachem Laboratories) as filter media to maximize the surface area and reduce impact on airflow due to settling. It is important to note that airflow is assessed after filtration through the carbon beads, so changes in airflow can be visualized and compensated for.

*Flow regulation*: Filtered air is divided into two rotameters (1–10 L/min; LZQ-7; Yuyao Shunhuan Flowmeter) to create primary and secondary supply lines that can be regulated independently. Nickel-plated brass connectors (4 mm tube by male 1/16″ BSPT; #6220N104; McMaster-Carr) were used to connect air lines.

**Figure 3.**
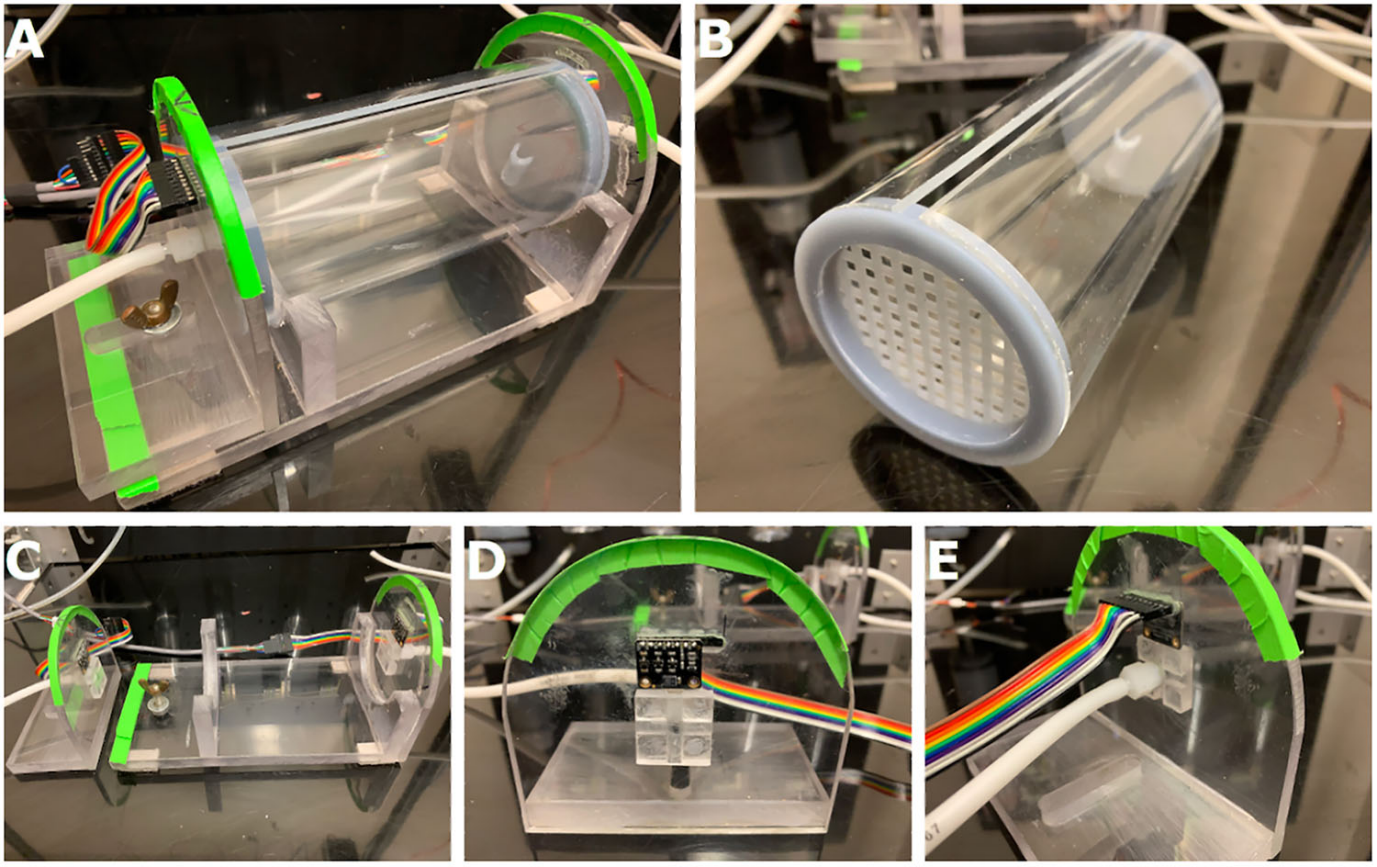
ODORS testing chamber assembly. ***A***, Testing chamber; secondary section is on the left, with the wingnut and adjustment slot visible. ***B***, Testing tube and end caps. Secondary side (perforated end cap) is at the close end, and the primary side (short odor port end cap) is at the far end. End caps are friction fit. The slot along the top allows air to escape and a tethered subject to be tested. ***C***, Separate primary (right) and secondary (left) sections of the holder; the screw and wingnut to attach the secondary section are visible on the left end of the primary section. ***D***, Inside of the secondary inlet, with the air diverter (25 × 25 × 6 mm acrylic with 3-mm-wide and 3-mm-deep slots facing the air inlet), VOC sensor, and VOC sensor header slot (with header, behind the sensor) visible. The primary section uses the same layout. ***E***, Outside of the secondary inlet, with the air line connection and VOC sensor header visible.

*Primary supply line*: The primary supply line provides stimulus air (clean or odorized) to the testing chamber (Figs. 1–3). From the rotameter, serial air supply branches are created using super-flow tee and 90° elbow connectors (1/8″ tube; #2808K166 and #2808K115; McMaster-Carr), with one branch per valve; valves include one clean air valve (single-tube, normally open, pinch solenoid valve; #360P021-42; NResearch) and four odorant valves (double-tube, normally closed, pinch solenoid valves; #648P031-42; NResearch); odorant valves used a double-tube design to isolate the inlet and outlet lines for each odorant bottle. Air passing through the clean air valve forms the start of a common downstream stimulus air feed to the testing chamber. For odorant valves, clean upstream supply air passes through the valve, enters the odorant bottle to mix with odorized stimulus air, then exits the odorant bottle, and passes back through the valve before joining the downstream stimulus air feed. On the downstream side, air from each valve is combined into the downstream stimulus air feed using wye connectors. By using the clean air valve as the start of the downstream stimulus air feed, the line is continually flushed with clean air between stimulus presentations to prevent cross-contamination of odor stimuli.

*Secondary supply line*: The secondary supply line is used to provide positive pressure to the testing chamber to localize stimulus air near the primary air inlet, allowing location-based scoring similar to the standard OHD task (Yang and Crawley, 2009) and improving clearance of odor stimuli during intertrial intervals.

*Odorant bottles*: Odorant bottles consist of clear 250 ml polyethylene terephthalate bottles, black polypropylene disc caps (24-410 size), and C-Flex tubing (Fig. 2). Caps are modified by removing the disc top to expose the opening (0.240″ in diameter) in the base of the cap; a second hole of similar size (∼0.25″) is drilled into the adjacent flat portion of the cap. Short sections of C-Flex tubing are threaded through each hole, with the portion at the top inserted into a solenoid pinch valve and connected to the upstream and downstream lines, allowing odorant bottles, caps, and tubing to be easily replaced as an entire unit to prevent odor cross-contamination.

#### Testing chamber

The testing chamber is composed of the testing tube, holder, and end caps.

*Testing tube*: The testing tube houses the subject during testing. For mice, this consists of a clear acrylic tube (2.75″ inner diameter, 6″ long) with a 0.125″ slot cut lengthwise along the top of the tube (Fig. 3*A*); this size allows subjects enough room to turn around, rear, and self-groom even with a tether attached. The slot also allows free movement of a recording tether and allows air to exit the testing tube. The tube is also removable for cleaning.

*Tube holder*: The holder provides a stable base and localizes the air inlets and volatile organic compound (VOC) sensors at either end of the tube (Fig. 3). A primary section contains testing tube supports and the primary air inlet and VOC sensor; a secondary section contains the secondary air inlet and VOC sensor and is used to clamp the testing tube into place. A slot in the secondary section fits over a screw mounted in the primary section, and a wingnut is used to clamp it down (Fig. 3). Small air diverters (0.25″ thick blocks with 0.125″ slots cut in the shape of a cross, facing the inlets) are mounted over each air inlet to diffuse incoming air. VOC sensors (CCS811 Air Quality Sensor; DFRobot) are mounted just above the diverters using 10-pin 2.54 mm pitch sockets mounted in slots (∼1″ long and 0.125″ tall). VOC sensors use a seven-pin 2.54 mm pitch male header, allowing adjustment of the sensor position above the inlet (Fig. 3).

**Figure 4.**
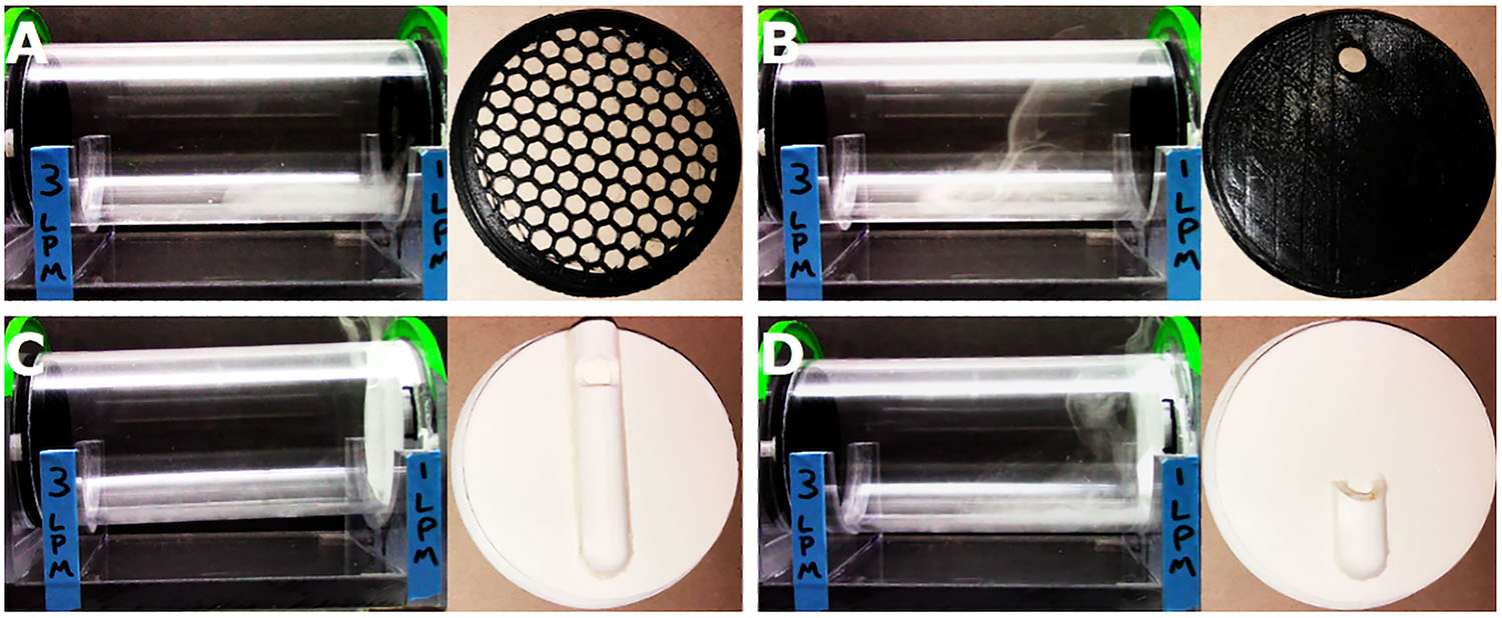
Airflow patterns in the ODORS testing chamber. Still images of testing chamber airflow patterns with glycerine-based “fog” using four different styles of end caps on the primary (right) side. ***A***, Perforated end cap resulting in minimal localization and diffuse airflow. ***B***, End cap with odor port resulting in localized source and focal stream of air. ***C***, End cap with long tube with odor port resulting in localized source with minimal air entering chamber. ***D***, End cap with short odor tube resulting in localized source and air primarily restricted to the primary side. All combinations used a perforated end cap on the secondary (left) side. Stills were taken 2–5 s after the fog entered the chamber. LPM, liters per minute.

*End caps*: End caps (Figs. 3, 4) were used at each end of the testing tube to (1) contain the subject when inserting or removing the testing chamber from the holder; (2) ensure that the slot along the top of the testing chamber does not pinch a recording tether; (3) prevent subjects from damaging the VOC sensors; and (4) direct airflow within the testing chamber. The primary end cap is solid with a single, short outlet tube designed to direct the airflow toward the slot in the top of the testing tube. Through visual observations of airflow patterns using a glycerine-based “fog,” we found that aiming airflow directly at the slot caused most of it to exit the chamber and resulted in relatively little odorized air remaining in the testing chamber (where it could reach the subject). As a result, we chose to direct the airflow slightly away from the slot to redirect some of the odorized air back into the testing chamber and ensure that a portion (but not all) of the odorized air reaches the subject; however, we did not assess subject performance based on the outlet type or airflow direction (i.e., either directly toward or adjacent to the slot), and this redirection may not be beneficial in all situations. The secondary end cap is perforated to allow even airflow from the secondary end of the testing tube. End caps were 3D printed with eSUN Hard-Tough Resin (Shenzhen Esun Industrial).

#### Electronics interface

The electronics interface consists of a valve control box and an Arduino Uno (Arduino). As testing systems can vary across labs, the overall design and use of these components rather than specific construction are discussed below.

*Valve control box*: The valve control box supplies power to activate the valves. It consists of an AC/DC power converter, a series of solid-state relays, input connections, and output connections. Input connections (in our case, a software-controlled multichannel digital input/output connector from our electrophysiology suite) control the relays, with valves connected to the output connections.

*Arduino Uno*: The Uno is connected to the VOC sensors and valve inputs on the valve control box and serves as an interface to monitor total VOC (tVOC) levels and valve activation. Separate five-connector cables are used to connect the Uno to the VOC sensors and the valve trigger inputs. A 9VDC power supply keeps the Uno constantly running to minimize warm-up time for the VOC sensors.

### ODORS function

*Function*: The ODORS delivers clean or odorized stimulus air to a testing chamber.

*Air supply*: Our pump is rated at ∼16 L/min (253 GPH), allowing us to run the pump at submaximal levels to reduce noise. Rotameter needle valves allow further control of airflow through the primary (∼1 L/min) and secondary (∼3 L/min) lines; these flow rates were chosen based on end cap airflow patterns (Fig. 4) and time needed to clear odorants from the testing chamber.

*Valves*: Valves are supplied from the primary air supply line and recombine into a single downstream stimulus air feed (Fig. 1). The clean air valve forms the start of the downstream stimulus air feed, and each odor valve output feeds into this stream (Fig. 1); this ensures that the downstream stimulus air line is continuously flushed by clean air during habituation and intertrial intervals to reduce odor cross-contamination. Air is only fed through a single valve at any time (i.e., either the clean air valve or one odorant valve) to keep a consistent flow rate and prevent dilution of stimulus air.

*Odorant bottles*: Clean, empty odorant bottles were tested for the presence of tVOCs relative to baseline air prior to use; only bottles that exhibited relatively low tVOCs (∼30–70 ppb) were used for testing. Odorant bottles were only used with a single valve and air line combination, with social odors only used with the final (fourth) odorant valve. The social stimulus bottle, cap, and air lines, as well as the final section of air line between Valve 4 and the testing chamber, were all replaced when switching between male and female social odors.

*End caps*: End cap design was selected based on airflow pattern in the testing chamber (Fig. 4). A visible aerosol was created using a “fog” machine and 10% mixture of glycerine and water and then captured in a 4 L container and connected between the primary air supply line and the primary side of the testing chamber. Airflow patterns were assessed by assembling the testing chamber with specific end cap combinations, turning on the air pump, and recording videos of the resulting airflow. End caps tested on the primary side included as follows: perforated; solid with a single 10 mm odor port near the top; solid with a vertical odor “tube” containing a 10 mm odor port near the top; and solid with a shortened vertical odor tube. Each combination used a perforated end cap on the secondary side. End cap 3D models are available as Extended Data.

*VOC sensors*: CCS811 environmental sensors (ScioSense) on a breakout board (CCS811 Air Quality Sensor; DFRobot) were used to assess tVOC levels of air entering the testing chamber. These sensors allow raw tVOC readings from 0 to 32,768 ppb at a minimum delay of 250 ms, although our maximum tVOC readings were capped at ∼29,000 ppb, likely due to saturation of the sensors. However, these sensors have been discontinued and replaced by the manufacturer with ENS160 environmental sensors; while the data presented herein are based on the CCS811 sensors, we have tested a combination ENS160 VOC sensor and AHT21 temperature/humidity sensor breakout board and found that the CCS811 and ENS160 sensors provide comparable sensitivity to VOCs (Extended Data Fig. 5-1). Libraries for breakout boards are available from the board retailer and/or the Arduino IDE Library Manager.

*Arduino Uno*: VOC data and valve activation were recorded using an Arduino Uno. Open-source header and source files were obtained from DFRobot and Keyestudio (Shenzhen KEYES Robot), and a custom Arduino sketch and Python script (see below, Code and data availability) were used to record tVOC levels, valve activation, and stimulus timing in a .csv file. Header and source files and basic Arduino sketches should be available from any manufacturer producing Arduino-compatible breakout boards based on the CCS811.

### Testing paradigm

A modified version of the olfactory habituation/dishabituation (OHD) task was used to test functionality of the ODORS; for details on the standard OHD task, see Yang and Crawley (2009). All procedures were performed in accordance with the Dalhousie University animal care committee regulations.

Twelve female and 12 male C57BL/6NCrl (strain #027; Charles River Laboratories) mice (20 weeks of age) were housed in groups of three in standard mouse cages (30 × 19 × 13 cm; Allentown Caging). A habituation day followed by a testing day was performed twice, 2 weeks apart. A towel was placed over the odorant board to reduce noise from the valves.

**Figure 5.**
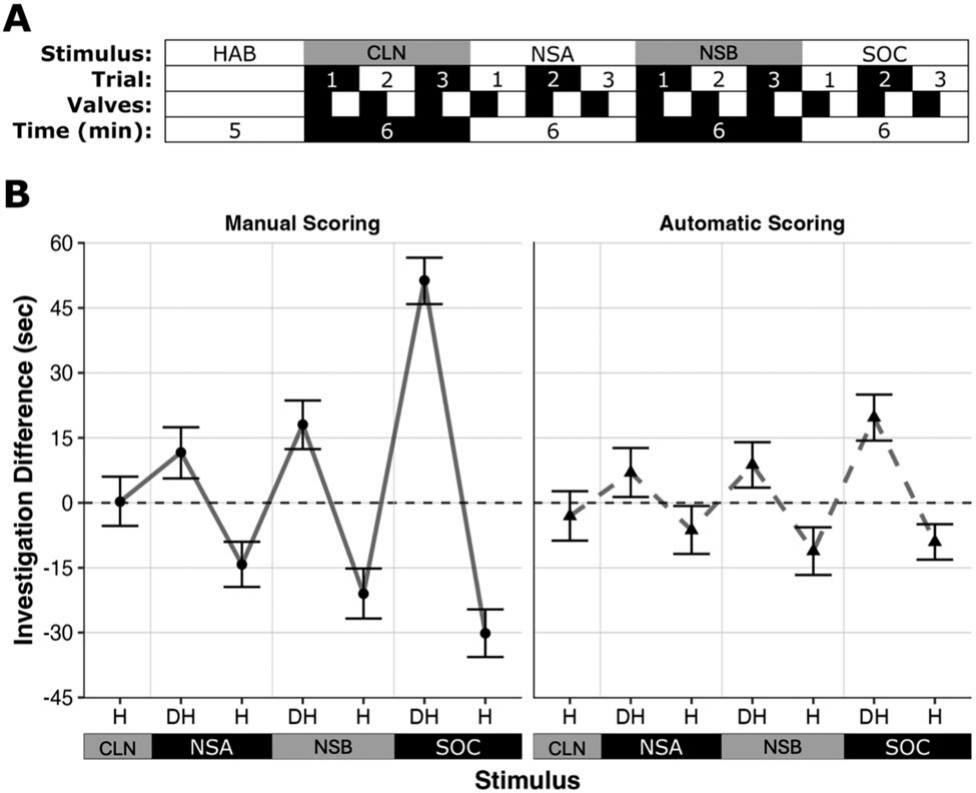
ODORS OHD timeline and overall results. ***A***, Timeline for testing sessions. Each session begins with habituation (HAB), followed by testing with the clean air control stimulus (CLN), nonsocial odor A (NSA), nonsocial odor B (NSB), and the social (same-sex urine) odor (SOC). Trials are shown below each stimulus. Valve activation during the session is shown below each trial; filled boxes indicate valve ON periods; empty boxes indicate valve OFF periods. The duration (in minutes) of each period (habituation and each of the four stimuli) is given at the bottom. ***B***, Automatic and manual scoring results for habituation (H; first to third presentation of one stimulus) and dishabituation (DH; third presentation of one stimulus to first presentation of the subsequent stimulus) effects (in seconds). Error bars indicate 95% CIs; points indicate median CI values. Values above 0 indicate increased investigation (dishabituation); values below 0 indicate decreased investigation (habituation). CLN, clean (control) stimulus; NSA, nonsocial Stimulus A; NSB, nonsocial Stimulus B; SOC, social (novel, same-sex urine) stimulus. tVOC readings from original sensor associated with behavior (CCS-811) and comparison with new (ENS-160) Arduino VOC sensors are shown in Extended Data Figure 5-1. Behavioral data visualized by sex, session, and odor set are shown in Extended Data Figure 5-2. VOC readings from sensor CCS-811 during behavioral testing are shown by cohort and session in Extended Data Figure 5-3.

For odorant presentation, we emulated the sequential, 2 min stimulus presentations from the standard OHD task by turning valves ON for 60 s, then OFF for 60 s, for each trial. When valves were ON, air passed through the respective odorant bottles to deliver stimulus air to the testing chamber; during the OFF period, clean air was supplied to flush out the lines and testing chamber. Our paradigm utilized one clean air control stimulus (Valve 1); two novel, nonsocial stimuli (Valves 2 and 3); and one novel social stimulus (Valve 4). Habituation to valve noise and air flow was done using six trials (12 min total) of control valve activation with the air pump on. For testing, subjects habituated to the testing chamber for 5 min with the air pump running, followed by 12 testing trials (three sequential presentations of each of the four stimuli; 24 min total); valves were triggered in the order listed above (Fig. 5).

10.1523/ENEURO.0161-25.2025.f5-1 Figure 5-1 **Total volatile organic compound (VOC) readings from original (CCS-811) and new (ENS-160) Arduino VOC sensors.** Total VOC levels (in ppb) for each stimulus and trial for original (CCS-811; dashed line) and new (ENS-160; solid line) Arduino-based VOC sensors. Readings were performed across a single 24 min session for each sensor. Sensors were tested consecutively using the same stimuli. Vertical dashed lines represent a change from one stimulus to the next. CLN = clean air (control) stimulus; NSA = non-social odour A; NSB = non-social odour B; SOC = social (novel, same-sex urine) stimulus. Download Figure 5-1, TIF file.

10.1523/ENEURO.0161-25.2025.f5-2 Figure 5-2 **ODORS olfactory habituation/dishabituation results by sex, session, and odour set.** Mean (+/- standard error) for investigation durations (in seconds) for each stimulus and trial based on **(A)** sex, **(B)** session, and **(C)** odour set. Vertical dashed lines represent a change from one stimulus to another. CLN = clean air (control) stimulus; NSA = non-social odour A; NSB = non-social odour B; SOC = social (novel, same-sex urine) stimulus; EA = ethyl acetate (monomolecular; non-social odour A); BAN = artificial banana (complex; non-social odour B); BDY = artificial brandy (complex; non-social odour A); BA = butyl acetate (monomolecular; non-social odour B). Download Figure 5-2, TIF file.

10.1523/ENEURO.0161-25.2025.f5-3 Figure 5-3 **ODORS olfactory habituation/dishabituation VOC readings by cohort and session.** Mean total volatile organic compound (tVOC) levels (in ppb) across sessions for each stimulus and trial for females (left) and males (right) for: **(A)** cohort 1, session 1; **(B)** cohort 2, session 1; **(C)** cohort 1, session 2; and **(D)** cohort 2, session 2. Values were calculated on a per-second basis but are presented per trial for clarity. Vertical dashed lines indicate start of each set of stimulus presentations. CLN = clean (control) odour; NSA = non-social odour A; NSB = non-social odour B; SOC = social (novel, same-sex urine) odour. Download Figure 5-3, TIF file.

Odor stimuli were ethyl acetate (#270989; Sigma-Aldrich), butyl acetate (#287725; Sigma-Aldrich), artificial brandy flavoring (McCormick), artificial banana flavoring (McCormick), and urine from novel, same-sex conspecifics. Banana, butyl acetate, and ethyl acetate odors have been used in previous olfactory studies (Bodyak and Slotnick, 1999; Yang and Crawley, 2009; Kass et al., 2013; Roddick et al., 2016; Oummadi et al., 2020), and ethyl acetate is present in brandy (Tsakiris et al., 2014), while butyl acetate is a component of artificial banana flavoring; as a result, ethyl acetate was paired with banana, and brandy was paired with butyl acetate to retain similar odor profiles (either ethyl acetate/brandy or butyl acetate/banana) across sessions while keeping stimuli distinct within a given session. We counterbalanced these odor combinations to assess behavior in response to both complex (e.g., banana, brandy) and monomolecular (e.g., butyl acetate, ethyl acetate) odor stimuli and to verify that our paradigm was repeatable in the same subject using novel odors. Our undiluted urine stimuli initially measured in the range of 10,000–13,000 ppb; thus, nonsocial odors were diluted with purified water to peak initial VOC concentrations in that range. Pooled urine stimuli were obtained from novel, sexually naive, same-sex conspecifics; to ensure novelty, the social stimulus used in the first session consisted of urine obtained from C57BL6/SJL mice (strain #100012; Jackson Laboratory, ME), while the social stimulus used in the second session was obtained from an outbred strain, Crl:CD1(ICR) mice (strain #022; Charles River Laboratories).

### Data analysis

#### Missing data

Second session data from two males was excluded; one was killed due to injuries from home-cage aggression prior to Session 2, and the other was tested but the video was not saved due to technical issues. No other data were excluded.

#### Scoring

Manual scoring to record the duration of investigation was performed from recorded videos; investigation was classified as any period in which the subject was facing the primary (stimulus) side of the testing chamber with its snout 2 cm or less from the end and not engaged in self-grooming. Manual scoring was performed blind. Automatic scoring was performed using Biobserve Viewer 3.0 (Biobserve). Pixel to real-world distances were calibrated in the program prior to scoring (0.458 mm/pixel). Raw pixel coordinates were imported into R (Version 4.2.2 “Innocent and Trusting”; The R Foundation for Statistical Computing) using RStudio (Version 2022.12.0 + 353; Posit Software) and used to assess location and movement direction of the subject within the testing chamber. Investigation was considered any period in which the subject's center of mass was between 44 pixels (2 cm) and 132 pixels (6 cm) from the primary side of the testing chamber during or after motion toward the primary end; the subject was assumed to be turning away from the stimulus if the body was <2 cm from the primary end of the testing chamber. Frames spent investigating were then converted to investigation time by dividing over the average number of frames per second. For manual and automatic scoring, timestamped investigation periods were compared with trial start and end times (obtained from valve onset/offset times) to determine total investigation period for each trial. VOC levels, stimulus type, and valve onset/offset were automatically recorded to CSV files at ∼260 ms intervals. VOC levels were recorded at both the primary and secondary inlets.

#### Statistical models

Data analysis was performed using mixed-effect models for the effect of stimulus and presentation number. To assess habituation (reduced investigation from the first to the final presentation of a single odor) and dishabituation (increased investigation from the final presentation of one odor to the first presentation of the subsequent odor), behavioral analysis was performed using only the first and third (i.e., final) presentation of each odor. Statistical models assessed main effects and the interaction of odor stimulus and presentation number (first or third) on investigation duration, with the subject as a random effect. Estimated marginal means were assessed post hoc using Holm–Bonferroni’s correction to examine directions of effects. Bootstrapped 95% CIs were calculated for the effect of stimulus and presentation on investigation duration.

#### Habituation/dishabituation

Preliminary assessment of manual scoring suggested that habituation/dishabituation effects were similar across sexes, sessions, and odor sets (Extended Data Fig. 5-2); as our goal was to validate the apparatus and associated paradigm, data were collapsed across sex, session, and odor set for manual and automatic scoring (*n* = 24 for first session; *n* = 22 for second session).

### Code and data availability

The Arduino and Python scripts and 3D end cap models described in the paper are freely available at https://github.com/FilipKosel/ODORS and are available as Extended Data. The Python script was run using the Anaconda interpreter on a PC running Windows 10. For the dataset associated with this paper, see https://doi.org/10.20383/102.0709.

10.1523/ENEURO.0161-25.2025.d1 Data 1 Download Data 1, ZIP file.

## Results

### End cap designs

Assessment of end cap design was based on visual observation of airflow patterns. Specifically, our aim was to emulate odor presentation of the traditional OHD task (9, 10) which utilizes a localized odor source (e.g., cotton swab with an odorant) and allows some diffusion of the odor without the use of air currents. We found that end cap designs resulted in distinct airflow patterns within the testing chamber (Fig. 4). The perforated design (Fig. 4*A*) and the odor port design (Fig. 4*B*) resulted in airflow patterns that spread lengthwise across the testing chamber. The long tube design resulted in a distinct stimulus source (the odor port) with minimal airflow into the testing chamber with the majority escaping through the testing chamber's tether slot (Fig. 4*C*). The short tube design resulted in a distinct stimulus source (the primary end of the odorant tube) and airflow localized to the primary end of the testing chamber (due to positive pressure from the secondary side; Fig. 4*D*). The short tube design was selected for use in the final apparatus.

### Investigation

Both manual and automatic scoring indicate that subjects exhibit habituation (decreased investigation of a repeated odor) and dishabituation (increased investigation to a novel odor) to nonsocial and social stimuli, with manual scoring indicating larger habituation/dishabituation effects than automatic scoring (Fig. 5).

For manual scoring, there was an effect of stimulus (*χ*^2^_(3)_ = 178.5980; *p* < 0.001), presentation (*χ*^2^_(1)_ = 89.8900; *p* < 0.001), and a stimulus by presentation interaction (*χ*^2^_(3)_ = 43.2160; *p* < 0.001). Post hoc analysis indicated that overall investigation of the social odor was higher than the control (*t*_(333.12)_ = 11.711; *p* < 0.001), first nonsocial odor (*t*_(333.12)_ = 10.617; *p* < 0.001), and second nonsocial odor (*t*_(333.18)_ = 10.036; *p* < 0.001). For presentation, overall investigation was higher for the first trial than the third trial (*t*_(333.16)_ = 9.461; *p* < 0.001). For the stimulus by presentation interaction, there was habituation for the first nonsocial odor (*t*_(333.18)_ = 3.775; *p* = 0.003), second nonsocial odor (*t*_(333.29)_ = 5.758; *p* < 0.001), and the social odor (*t*_(333.06)_ = 9.256; *p* < 0.001); there was no habituation to the clean air control stimulus. There was also dishabituation from the first to the second nonsocial odor (*t*_(333.18)_ = 5.172; *p* < 0.001) and from the second nonsocial odor to the social odor (*t*_(333.29)_ = 14.508; *p* < 0.001).

For automatic scoring, there was an overall effect of stimulus (*χ*^2^_(3)_ = 46.2371; *p* < 0.001), presentation (*χ*^2^_(1)_ = 5.6925; *p* = 0.017), and a stimulus by presentation interaction (*χ*^2^_(3)_ = 8.1309; *p* = 0.043). For stimulus, overall investigation of the social odor was higher than the control (*t*_(161)_ = 6.031; *p* < 0.001), first nonsocial odor (*t*_(161)_ = 4.997; *p* < 0.001), and second nonsocial odor (*t*_(161)_ = 5.432; *p* < 0.001). For presentation, overall investigation was higher for the first trial than the third trial (*t*_(161)_ = 2.386; *p* = 0.018). For the stimulus by presentation interaction, there was habituation for the social odor (*t*_(161)_ = 3.189; *p* = 0.038). There was also dishabituation from the second nonsocial odor to the social odor (*t*_(161)_ = 6.380; *p* < 0.001). Investigation duration was not related to VOC levels, as subjects exhibited high levels of investigation of social stimuli even with relatively low VOC levels (Extended Data Figs. 5-2, 5-3).

## Discussion

Here, we discuss the design, assembly, and testing of the ODORS, an economical apparatus that can efficiently and accurately test behavioral responses to odor stimuli in tethered and untethered rodents and that can be easily modified and integrated with existing testing systems. This system can be modified to use any number of odor stimuli by adding/subtracting odorant valves and bottles, and timing of valve onset/offset can be controlled from the Arduino itself; as a result, the ODORS can be used as a stand-alone unit or be directly controlled by other software. The ODORS OHD paradigm mimics the standard OHD task by allowing successive presentations of odor stimuli at defined intervals with an initial high odor concentration around the stimulus source followed by gradual dissipation over the course of the trial (Extended Data Fig. 5-3); we achieved this by alternating odorized air with clean air, and the timing and number of trials per stimulus (three 2 min trials) match existing OHD paradigms. The primary end cap was the short odor tube design (Figs. 3, 4), resulting in localized stimulus presentation from a distinct source (the odor tube) and allowing location-based scoring similar to existing OHD paradigms. Finally, results from the ODORS exhibit a habituation/dishabituation pattern similar to the standard OHD task (Fig. 5), suggesting that these two paradigms are functionally similar. Manual scoring also indicates that males and females show similar levels of investigation, that the test can be reliably performed twice in the same animals, and that investigation is largely similar across odor sets regardless of whether odorants are monomolecular or complex (Extended Data Fig. 5-1).

While the ODORS and the standard OHD task are functionally similar, the use of automated stimulus delivery and VOC sensors provide a number of additional benefits. Automated stimulus delivery reduces labor requirements, variability, and potential stress due to experimenter presence. Additionally, precise control of stimulus onset and offset removes any unintentional variability in trial duration or intertrial interval that may arise with manual stimulus presentation and allows synchronization of other data with stimulus presentation. Importantly, video recordings can be synchronized with stimulus delivery on a frame-by-frame basis for fully automatic scoring. Additionally, the apparatus has been designed for use with tethered and untethered subjects by the inclusion of a slot along the top of the testing chamber allowing for free movement of a tether (Kosel et al., 2024). Timing of stimulus delivery and VOC level thresholds can be used in real time to trigger electrical or optogenetic stimulation or during later analyses to identify periods of interest. We suggest that the ODORS will simplify and encourage olfactory research in rodent models to improve our understanding of this important modality.

## Synthesis

Reviewing Editor: Jibran Khokhar, Western University Department of Anatomy and Cell Biology Decisions are customarily a result of the Reviewing Editor and the peer reviewers coming together and discussing their recommendations until a consensus is reached. When revisions are invited, a fact-based synthesis statement explaining their decision and outlining what is needed to prepare a revision will be listed below. The following reviewer(s) agreed to reveal their identity: Mark Laubach, Manu Madhav.

Summary This manuscript presents a well-documented, open-source odorant delivery system (ODORS) designed for behavioral studies in rodents. The system supports both tethered and untethered setups, is modular for integration with other testing platforms, and is validated using VOC sensors and an olfactory habituation/dishabituation (OHD) task in mice. The authors provide design files, parts lists, and software, contributing a valuable tool for the behavioral neuroscience community. While the manuscript is clear and thorough overall, there are a few critical issues and several minor concerns that should be addressed to enhance reproducibility and clarity.

Major Comments

Sensor Availability and Compatibility: The air quality sensor used in the system has been discontinued, which limits replicability. Although the authors mention an alternative (line 202), they should validate that their hardware and software are compatible with this alternative and update their documentation accordingly. Including performance comparison data between the discontinued and replacement sensors is essential for transparency and reproducibility. The authors should also discuss other potential sensor options and regional vendor availability to ensure long-term sustainability of the system.

Assembly Instructions: Step-by-step images of the ODORS assembly process should be included in the extended data. While different labs may have unique setups (line 157), standardized instructions would greatly aid reproducibility and broader adoption.

Statistical Reporting Clarity: The methods section lacks detail on the statistical models used, particularly the mixed-effects models. The degrees of freedom and associated test statistics (e.g., chi-square and t-values) are unclear, especially given the reported sample size of 24 mice. The sex effects noted in Figures 5-1 and 5-2 need more thorough explanation, with clear connection to the statistical methodology.

Minor Comments

Abstract (lines 10-11): Improve flow by splitting the sentence: "...assess olfactory perception, memory, and motivation. However, these tasks require manual stimulus presentation..."

Line 96-97: Further explain the function of the carbon beads, particularly their impact on airflow.

Line 133: Sentence clarity: "...even with a tether attached. The slot also allows free movement of a recording tether..."

Line 150-151: Explain the purpose of directing airflow at a 5{degree sign} angle in the end cap design.

Line 209-210: Specify which breakout board and software library were used with the sensor.

Line 272-273: Outliers should be visualized in plots (e.g., Figures 5, 5-1) for transparency.

Figure 4: Improve contrast and clarity-fog patterns are hard to discern, especially in Fig. 4C which appears overexposed. Clarify why a short tube design with localized airflow is beneficial. Section 3.2: Include sample sizes and degrees of freedom for each statistical test.

Figure 1: Clarify airflow direction and outlet location. Fig. 1B shows two inlets-if both provide positive pressure, where does the air exit?

Figure 5A: Improve readability-use a different background (e.g., gray) for CLN and NSB blocks.

Spelling Consistency: Standardize on either British ("odour") or American ("odorized") spelling throughout.

Extended Data: In addition to STL files, provide editable 3D models (e.g., DWG, SLDPRT) for easier modification by users.
