# Supplementary figures and images for: The Odor Delivery Optimization Research System (ODORS): An Open-Source Olfactometer for Behavioral Assessments in Tethered and Untethered Rodents
Source: eNeuro. 2025 Dec 17;12(12):ENEURO.0161-25.2025. doi: 10.1523/ENEURO.0161-25.2025 (PMC12757508; doi:10.1523/ENEURO.0161-25.2025)

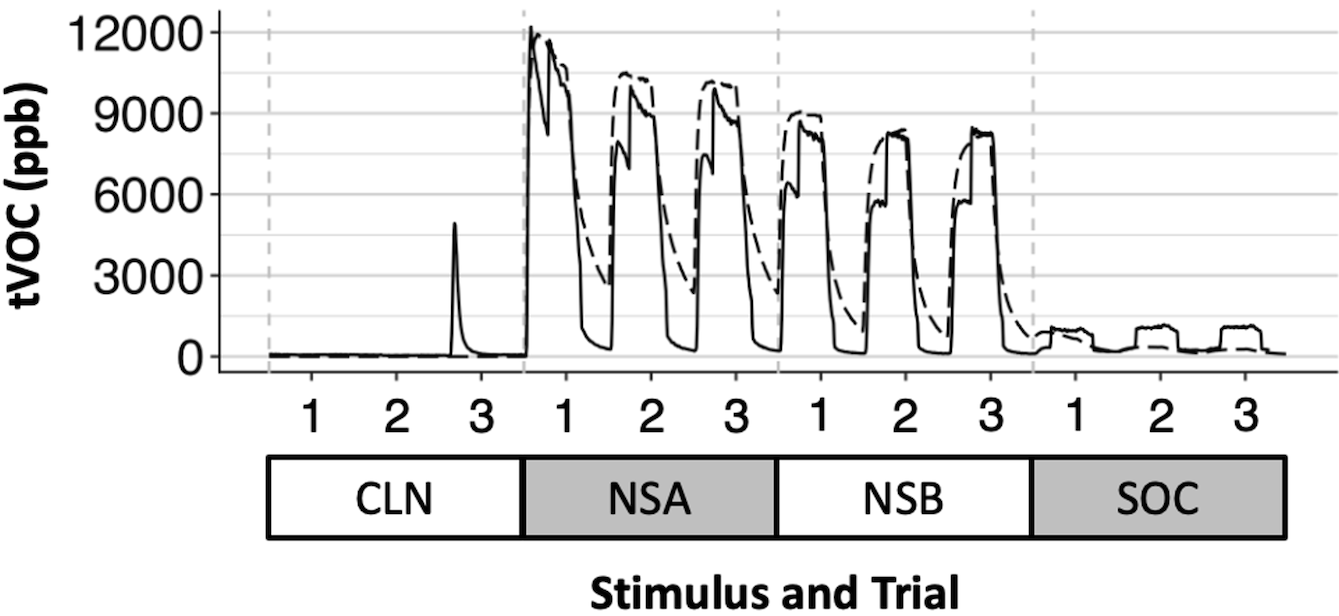

Supplement: Figure 5-1 — Total volatile organic compound (VOC) readings from original (CCS-811) and new (ENS-160) Arduino VOC sensors. Total VOC levels (in ppb) for each stimulus and trial for original (CCS-811; dashed line) and new (ENS-160; solid line) Arduino-based VOC sensors. Readings were performed across a single 24 min session for each sensor. Sensors were tested consecutively using the same stimuli. Vertical dashed lines represent a change from one stimulus to the next. CLN = clean air (control) stimulus; NSA = non-social odour A; NSB = non-social odour B; SOC = social (novel, same-sex urine) stimulus. Download Figure 5-1, TIF file. [file eneuro-12-ENEURO.0161-25.2025-s003.tif]

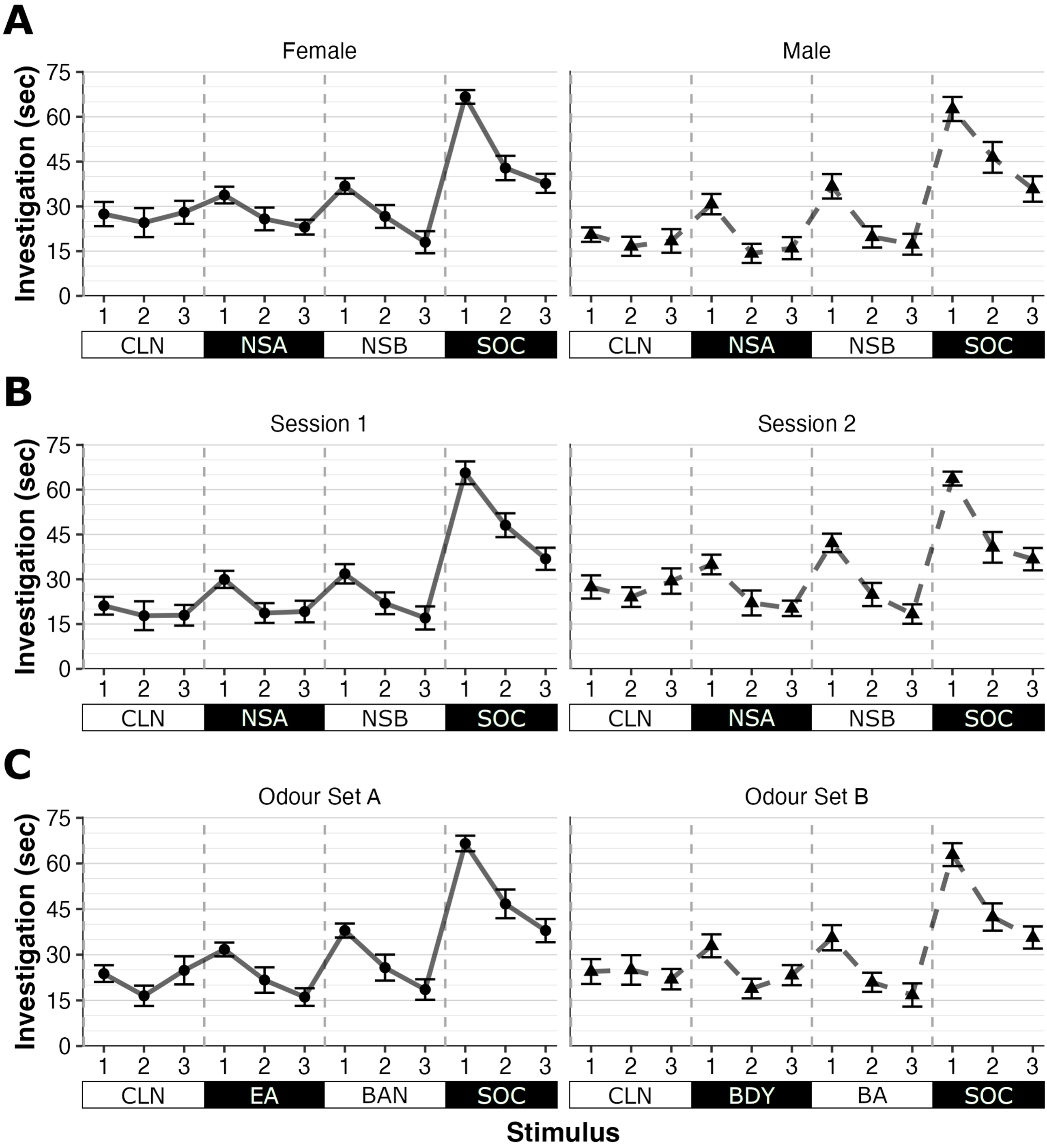

Supplement: Figure 5-2 — ODORS olfactory habituation/dishabituation results by sex, session, and odour set. Mean (+/- standard error) for investigation durations (in seconds) for each stimulus and trial based on (A) sex, (B) session, and (C) odour set. Vertical dashed lines represent a change from one stimulus to another. CLN = clean air (control) stimulus; NSA = non-social odour A; NSB = non-social odour B; SOC = social (novel, same-sex urine) stimulus; EA = ethyl acetate (monomolecular; non-social odour A); BAN = artificial banana (complex; non-social odour B); BDY = artificial brandy (complex; non-social odour A); BA = butyl acetate (monomolecular; non-social odour B). Download Figure 5-2, TIF file. [file eneuro-12-ENEURO.0161-25.2025-s004.tif]

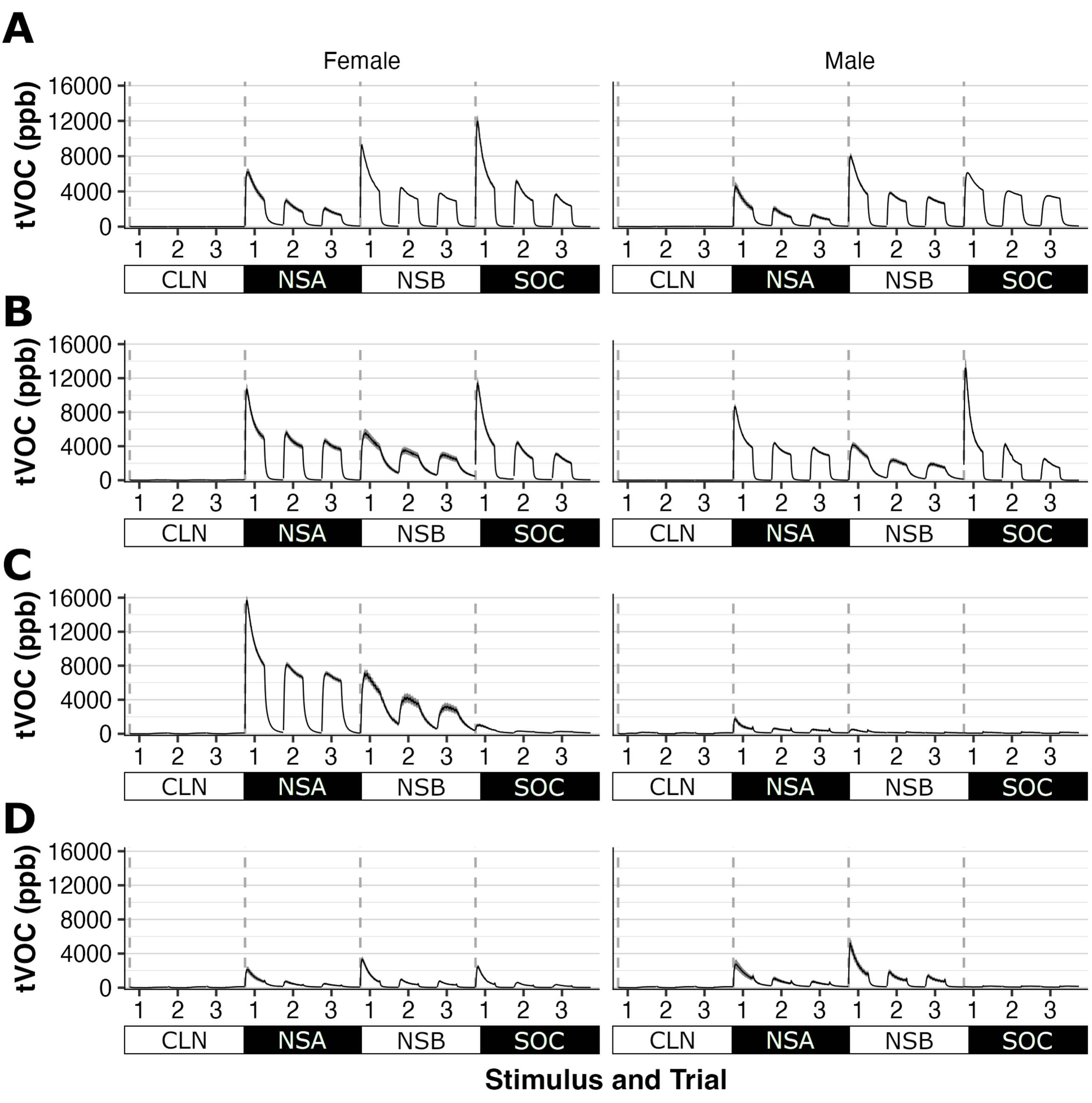

Supplement: Figure 5-3 — ODORS olfactory habituation/dishabituation VOC readings by cohort and session. Mean total volatile organic compound (tVOC) levels (in ppb) across sessions for each stimulus and trial for females (left) and males (right) for: (A) cohort 1, session 1; (B) cohort 2, session 1; (C) cohort 1, session 2; and (D) cohort 2, session 2. Values were calculated on a per-second basis but are presented per trial for clarity. Vertical dashed lines indicate start of each set of stimulus presentations. CLN = clean (control) odour; NSA = non-social odour A; NSB = non-social odour B; SOC = social (novel, same-sex urine) odour. Download Figure 5-3, TIF file. [file eneuro-12-ENEURO.0161-25.2025-s005.tif]
