# Supplementary material for: The Odor Delivery Optimization Research System (ODORS): An Open-Source Olfactometer for Behavioral Assessments in Tethered and Untethered Rodents
Source: eNeuro. 2025 Dec 17;12(12):ENEURO.0161-25.2025. doi: 10.1523/ENEURO.0161-25.2025 (PMC12757508; doi:10.1523/ENEURO.0161-25.2025)
Supplement: Data 1 — Download Data 1, ZIP file. [file eneuro-12-ENEURO.0161-25.2025-s001.zip › ODORS-main/ODORS-Assembly_Guide.pdf]

A step-by-step photo guide to assembling the olfactometer.

Hartley, Mackenzie, and Kosel, Filip - Oct 2025

Preview of Figures:

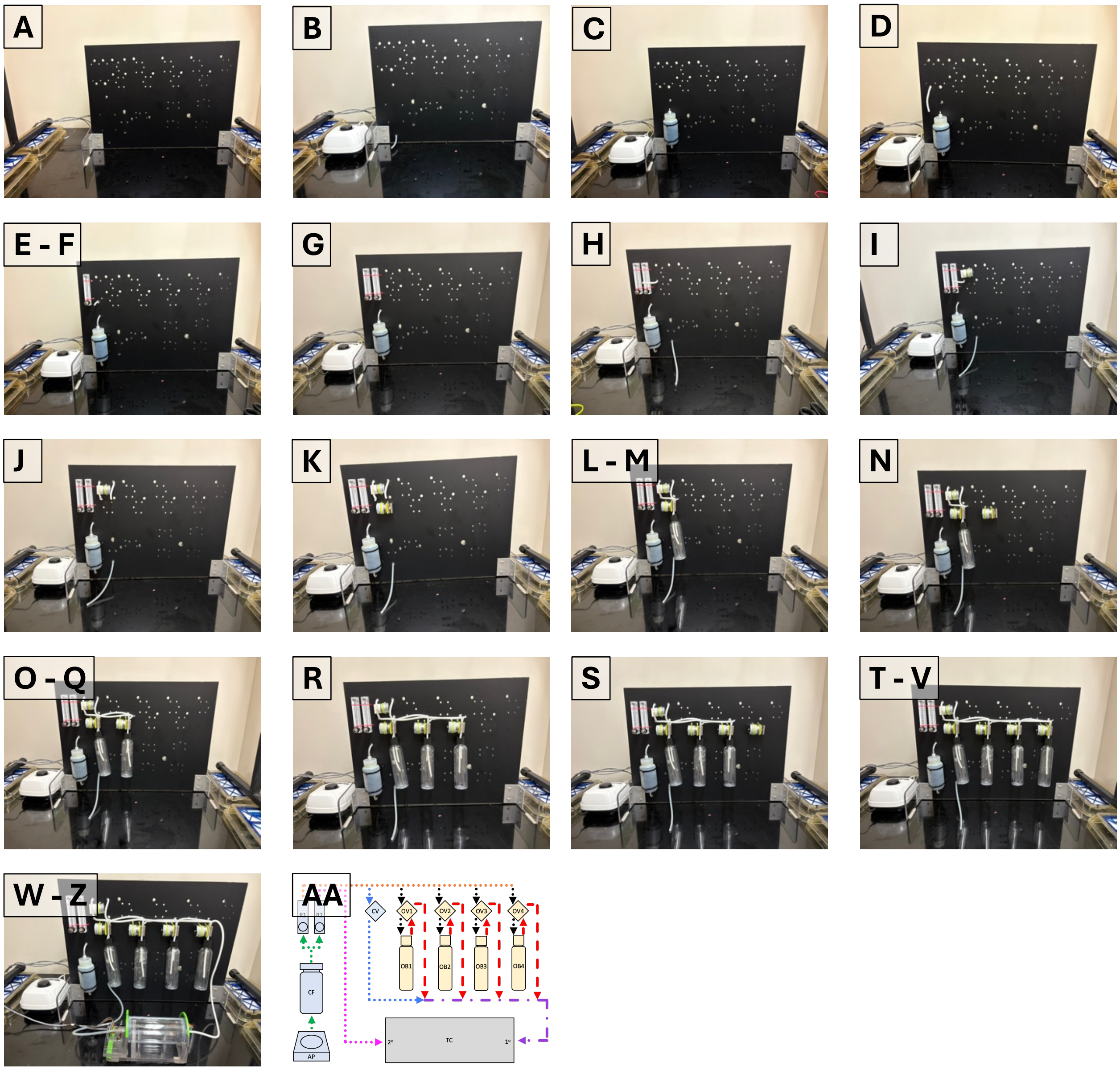

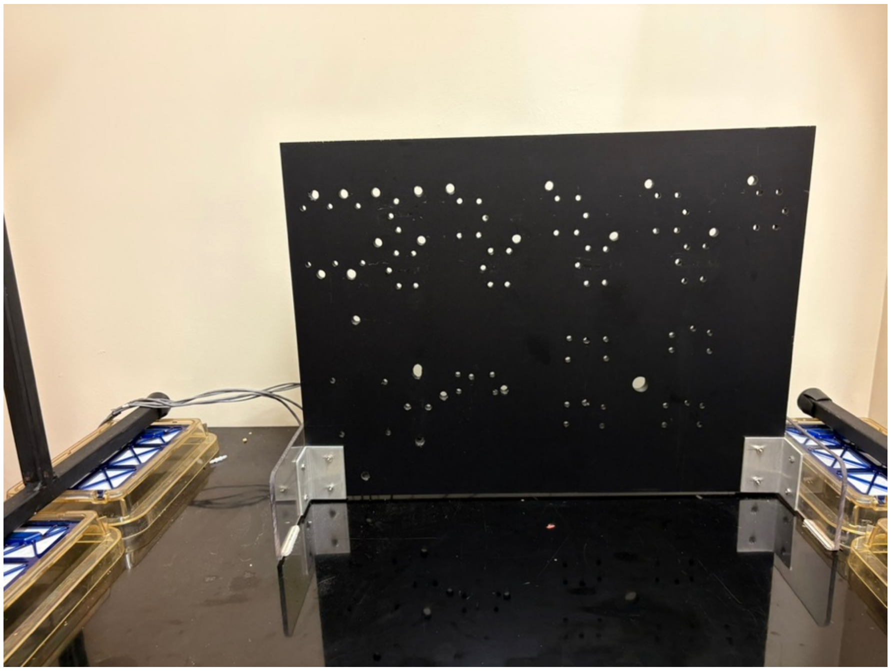

A) Plan component locations and create holes needed to attach all parts of the olfactometer to the odorant board. The number of stimuli, size of the board, and your physical space will determine the best location for each component; we recommend planning out component locations before drilling holes.

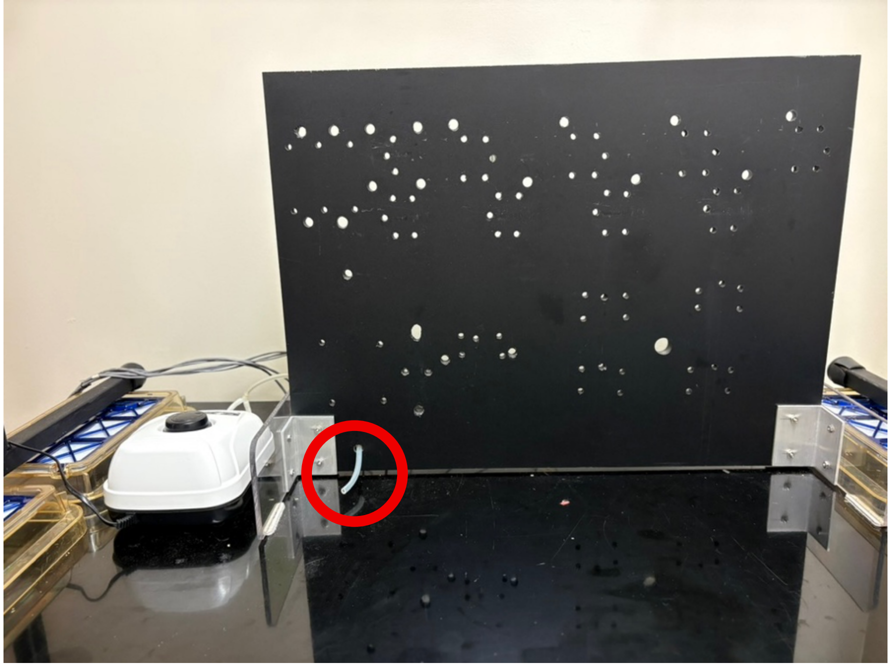

B) Place *air pump* beside the odorant board and run tubing from *air pump* to the odorant board, exiting out the front.

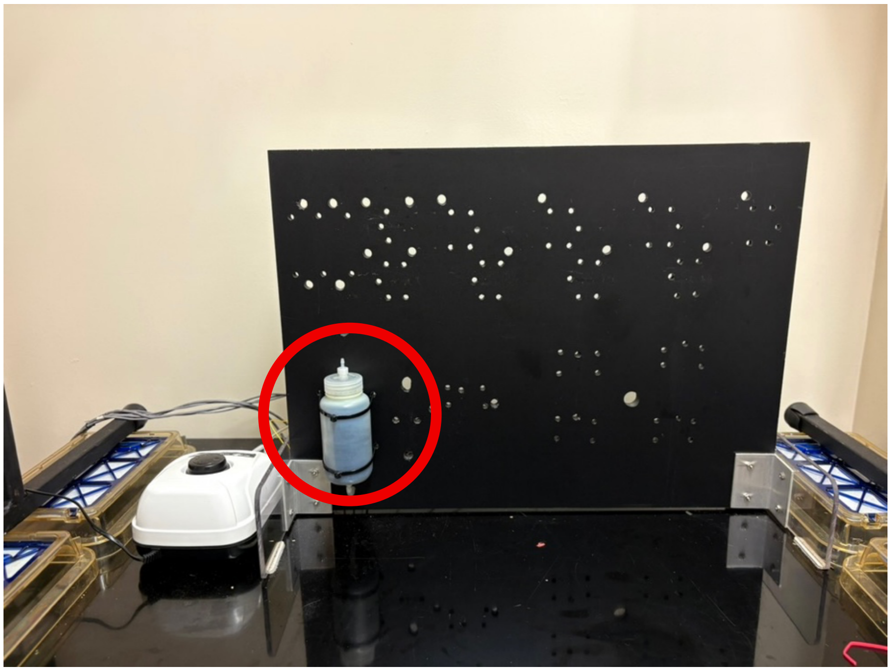

C) Connect the tube from the *air pump* to the bottom of the *carbon filter* and attach the *carbon filter* to the odorant board. Connect tubing to the top of the *carbon filter* and run it (from front to back) through the corresponding hole in the odorant board.

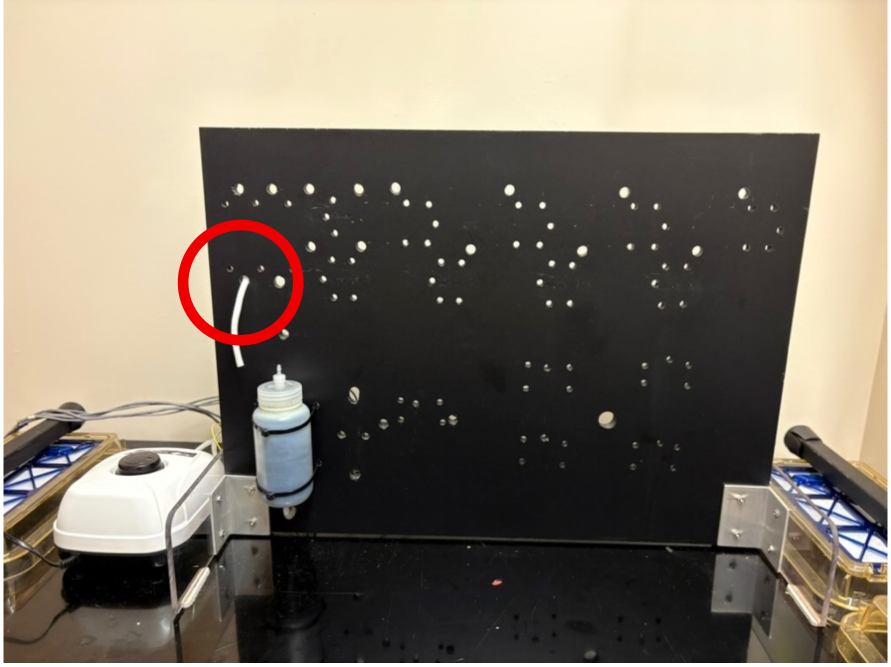

D) From the back of the board, connect the tube from the *carbon filter* to the bottom of a wye connector. Connect one tube to the top of the wye connector and pass it to the front of the board where the *primary rotameter* will go.

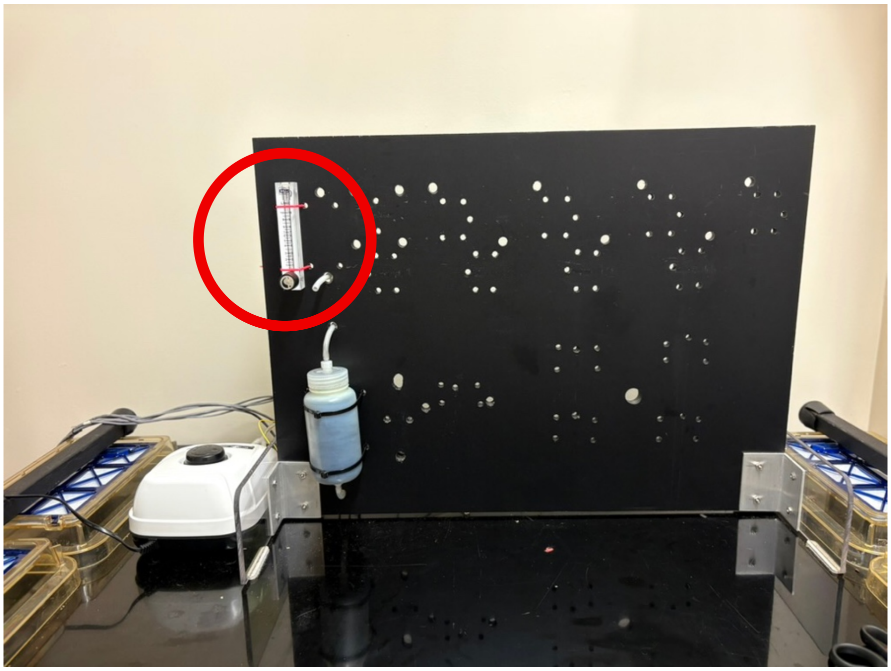

E) Connect the tube to the bottom (inlet) of the *primary rotameter* and attach the *primary rotameter* to the odorant board. Note that air can only flow in one direction (bottom to top) through the rotameters.

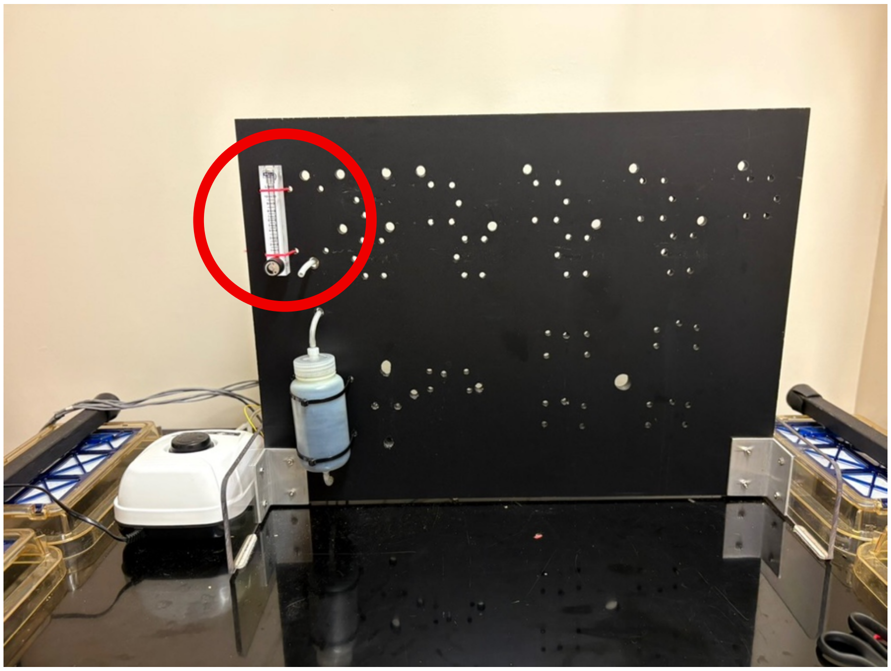

F) Connect a second tube to the top of the wye connector (see **Step D**) and pass it through the board for the *secondary rotameter*.

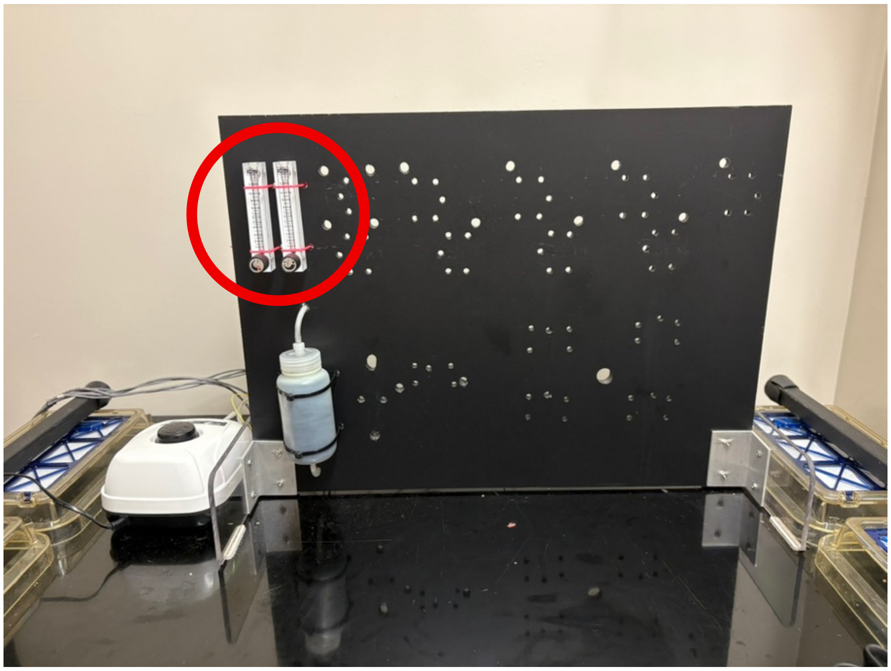

G) Connect the tube from **Step F** to the bottom (inlet) of the *secondary rotameter* and attach the *secondary rotameter* to the odorant board.

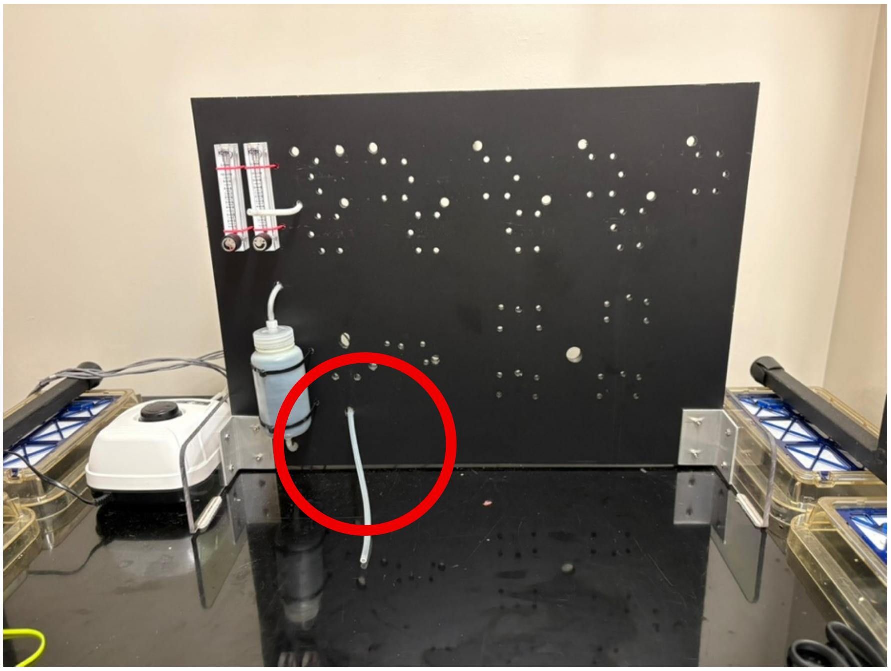

H) Connect a tube to the top (outlet) of the *secondary rotameter* and pass it to the front of the board near the bottom to form the *secondary air feed line*; ensure this tube is long enough to reach the *testing chamber*.

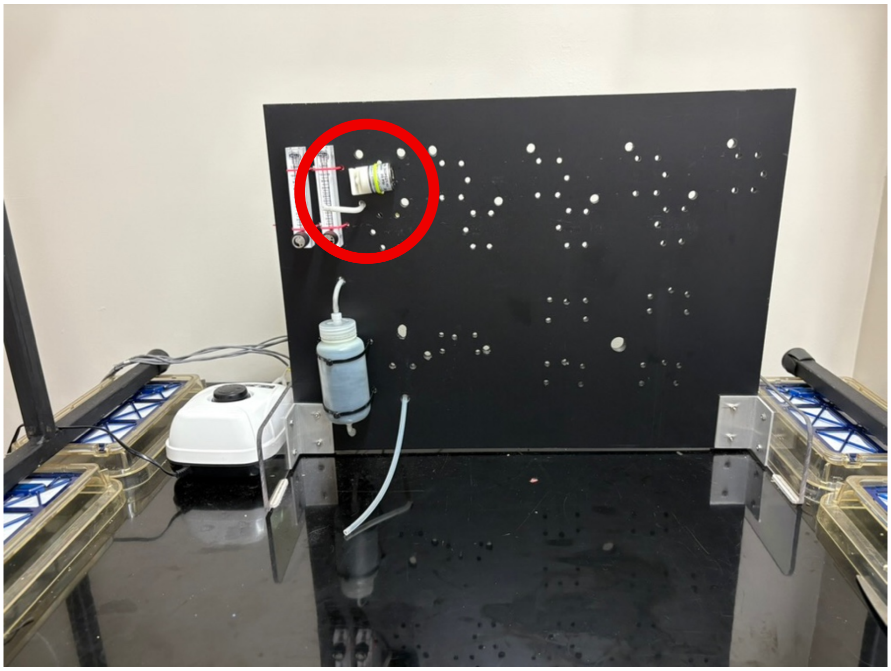

I) Attach the *clean air valve* to the board.

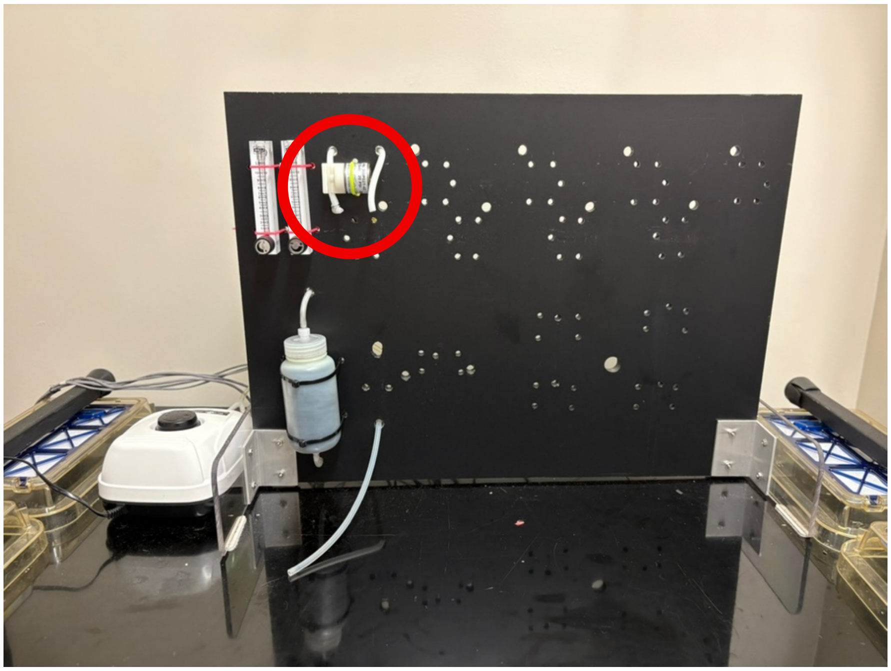

J) Attach a wye connector to the top (outlet) of the *primary rotameter* to form the start of the *primary air supply line*. Attach two additional tubes to the wye connector and pass them to the front of the board. Insert one tube into the *clean air valve* to form the *clean air feed branch*. The remaining tube is the *primary air supply line*. Tee connectors will be attached serially to the *primary air supply line* to create separate, parallel *odorant bottle supply branches* for each *odorant valve* and *odorant bottle* combination.

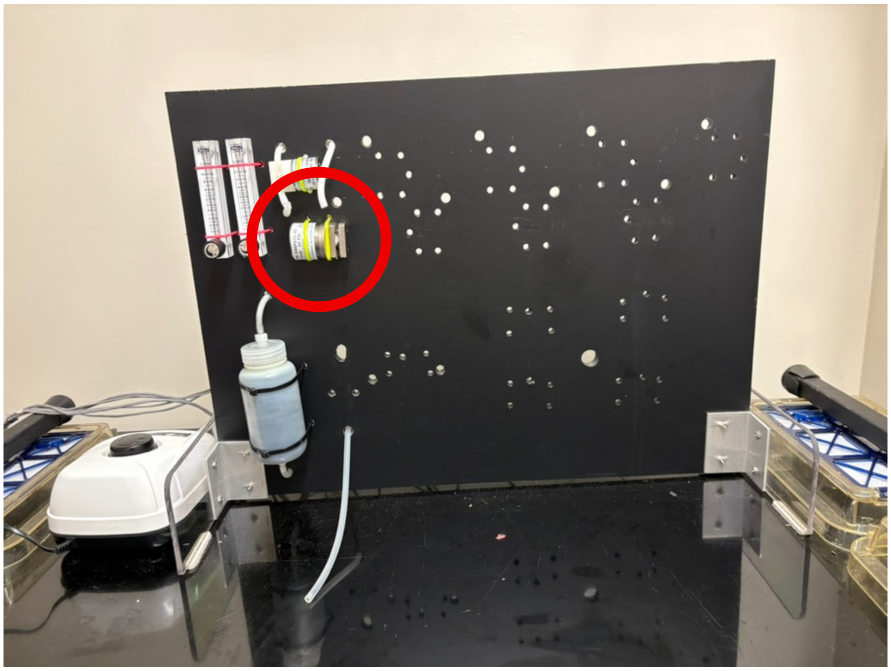

K) Attach the first *odorant valve* to the board.

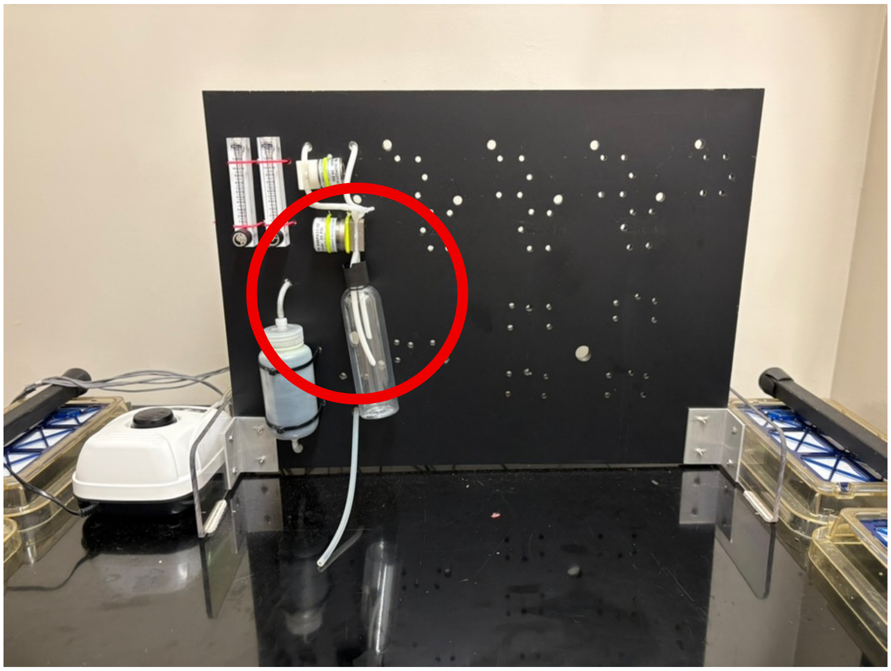

L) Attach a tee connector to the *primary air supply line* (**Step J**), then attach two tubes to the tee connector. One of these tubes will continue the *primary air supply line*. The other will become the first *odorant bottle supply branch*. Insert the first *odorant bottle supply branch* into the first *odorant valve* and then into the first *odorant bottle*.

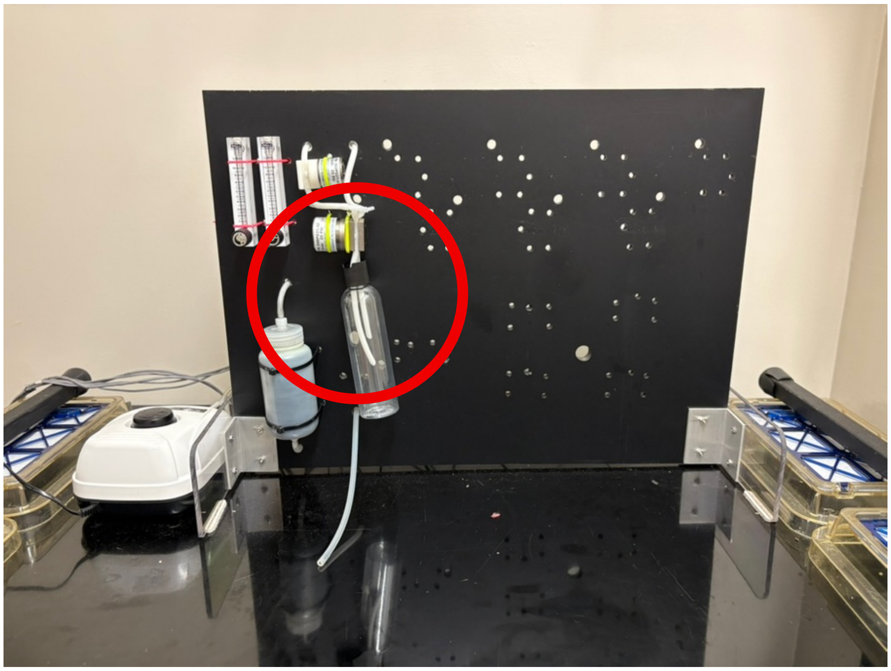

M) Insert a second tube into the first *odorant bottle*; this is the first *odorized air feed branch*. Insert the first *odorized air feed branch* into the first *odorant valve* and use a wye connector to join the first *odorized air feed branch* to the *clean air feed branch* (**Step J**). Attach a short section of tubing to the third leg of the wye connector; this will become the *stimulus air feed line*.

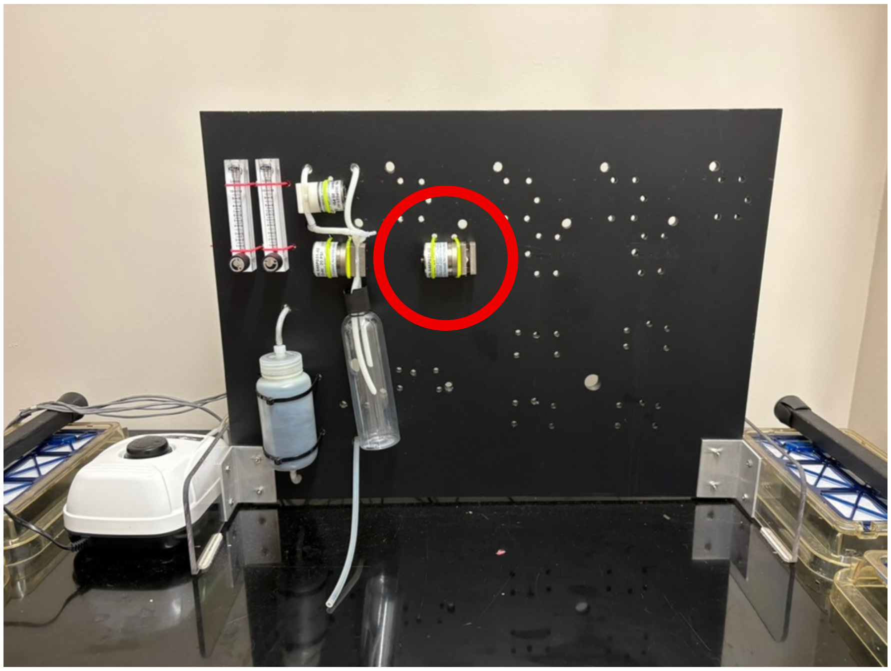

N) Attach the next *odorant valve* to the board.

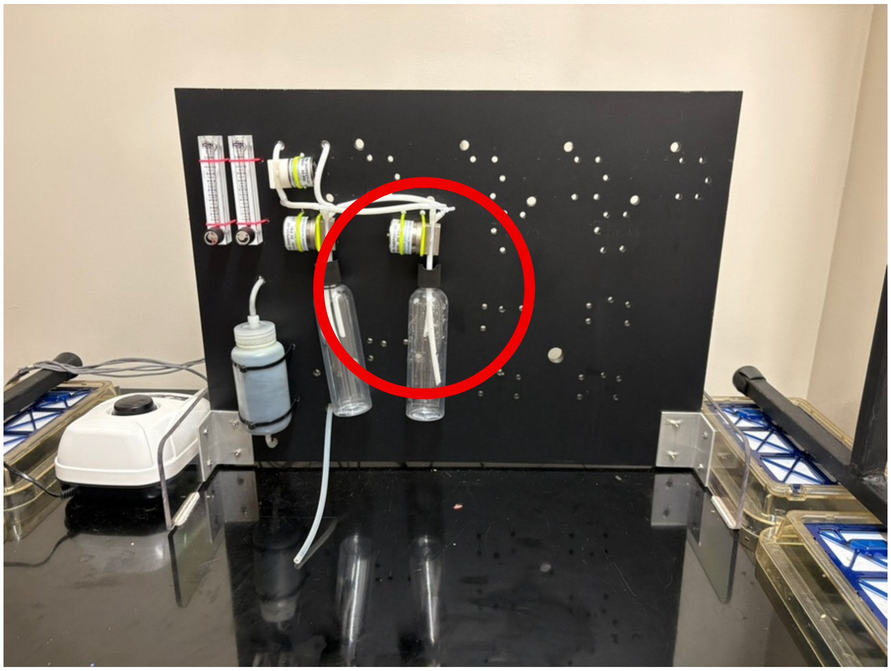

O) Attach a tee connector to the *primary air supply line*. Attach two additional pieces of tubing to the tee connector to continue to *primary air supply line* and create a new *odorant bottle supply branch*.

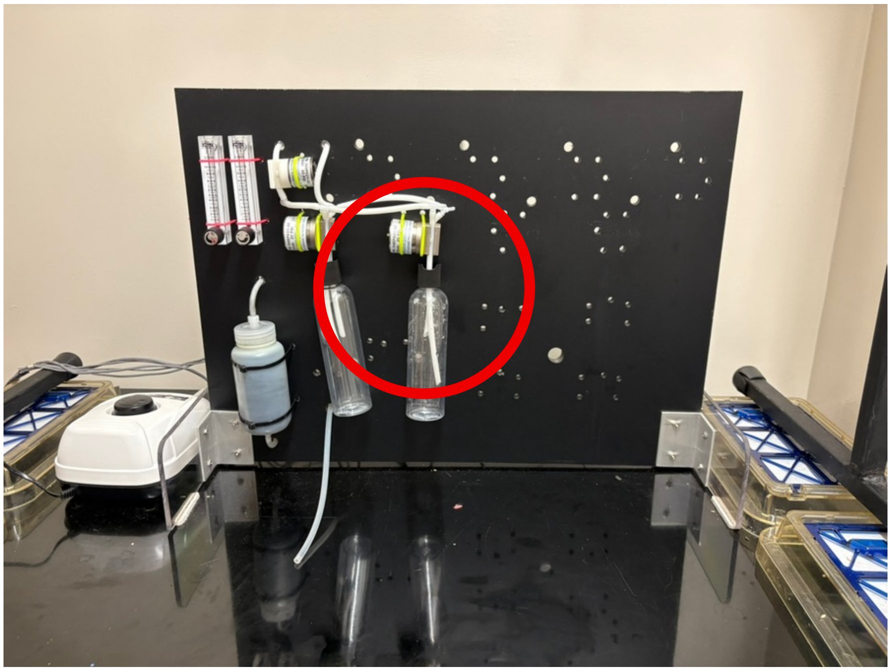

P) Insert the free *odorant bottle supply branch* into the next *odorant valve* and then into the corresponding *odorant bottle*.

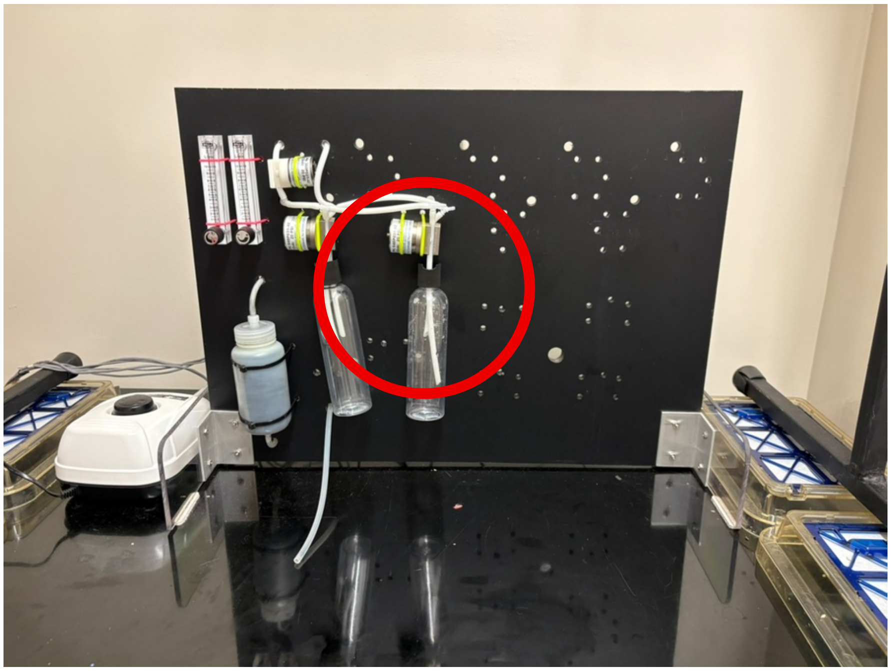

Q) Insert a free section of tubing into the *odorant bottle* and then into the corresponding *odorant valve* to form the next *odorized air feed branch*. Use a wye connector to combine the *odorized air feed branch* into the *stimulus air feed line*. Attach a third section of tubing to the wye connector to create the next section of the *stimulus air feed line*.

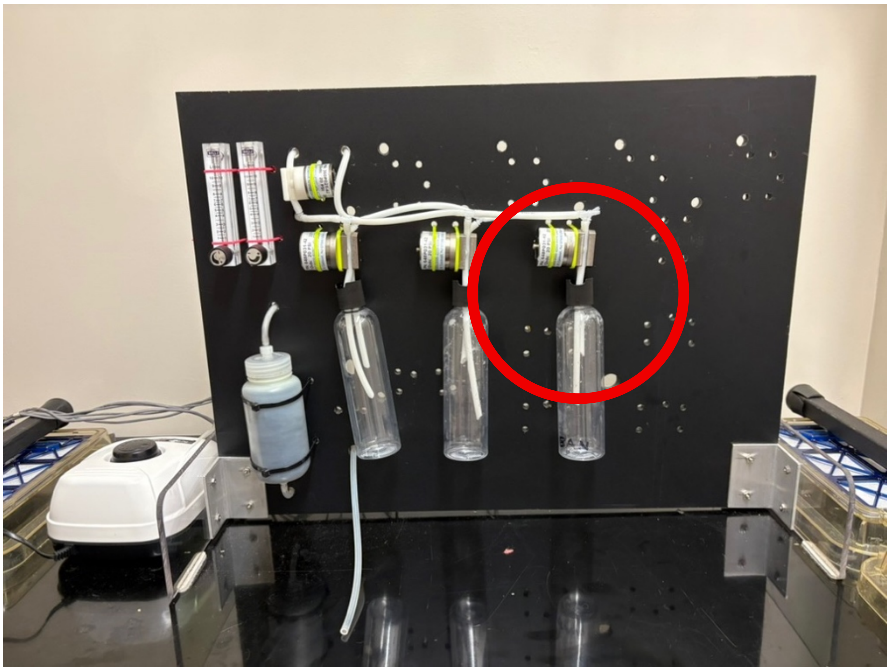

R) Repeat **Steps N to Q** for each additional *odorant valve* and *odorant bottle* combination, then continue to **Step S** for the final combination.

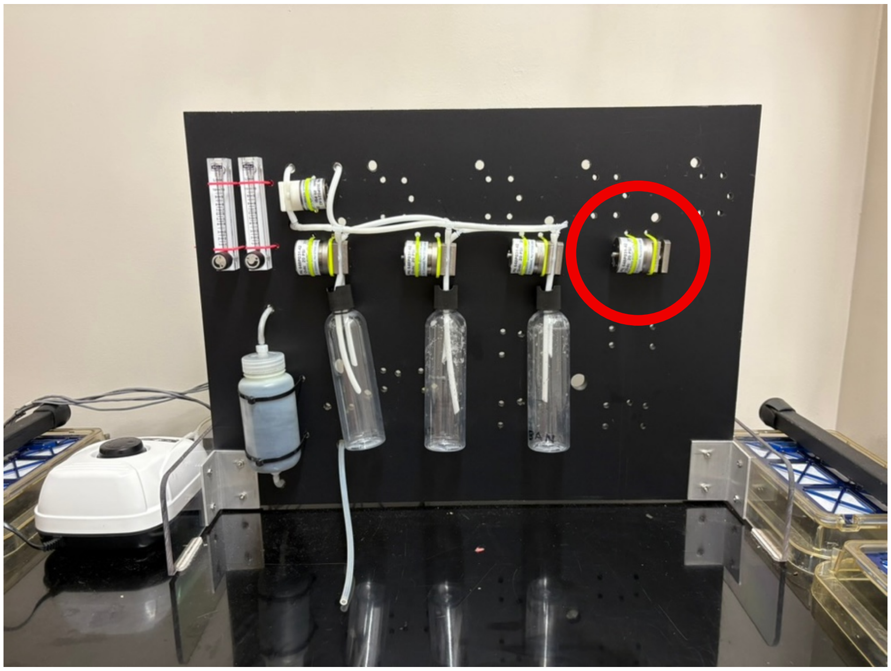

S) Attach the final *odorant valve* to the board.

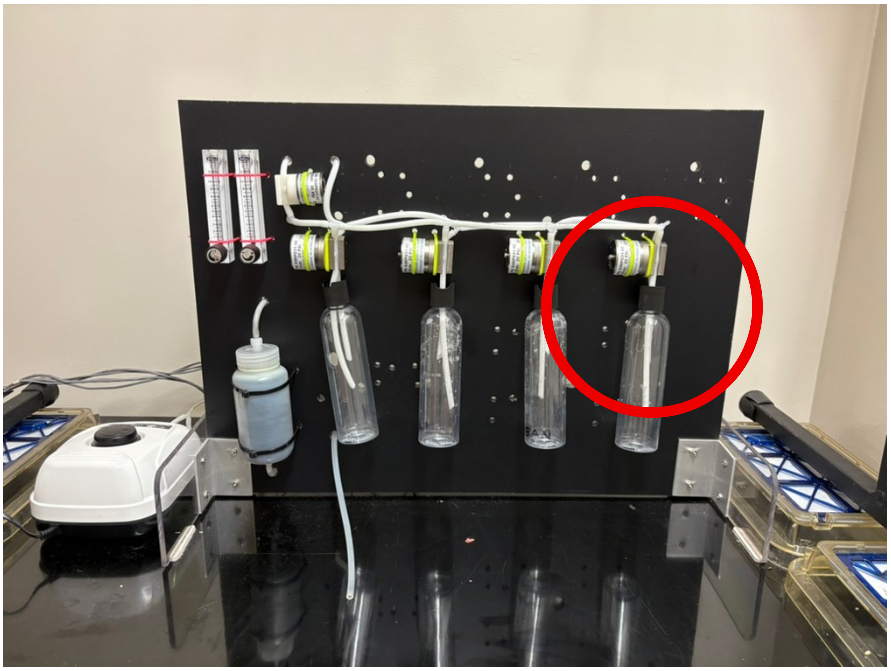

T) Attach a 90˚ connector to the *primary air supply line* to terminate the *primary air supply line* and create the last *odorant bottle supply branch*.

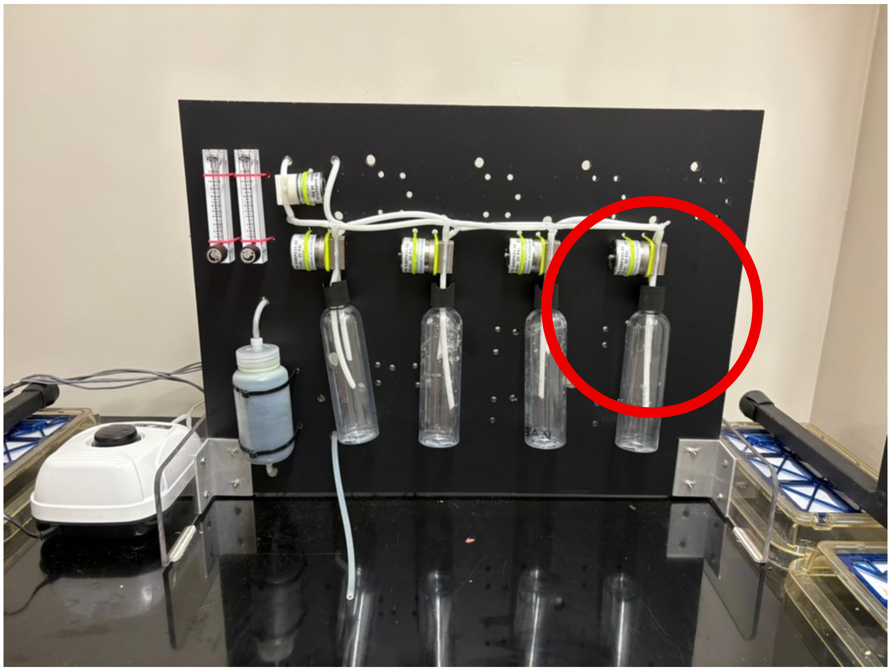

U) Insert the free *odorant bottle supply branch* into the final *odorant valve* and then into the corresponding *odorant bottle*.

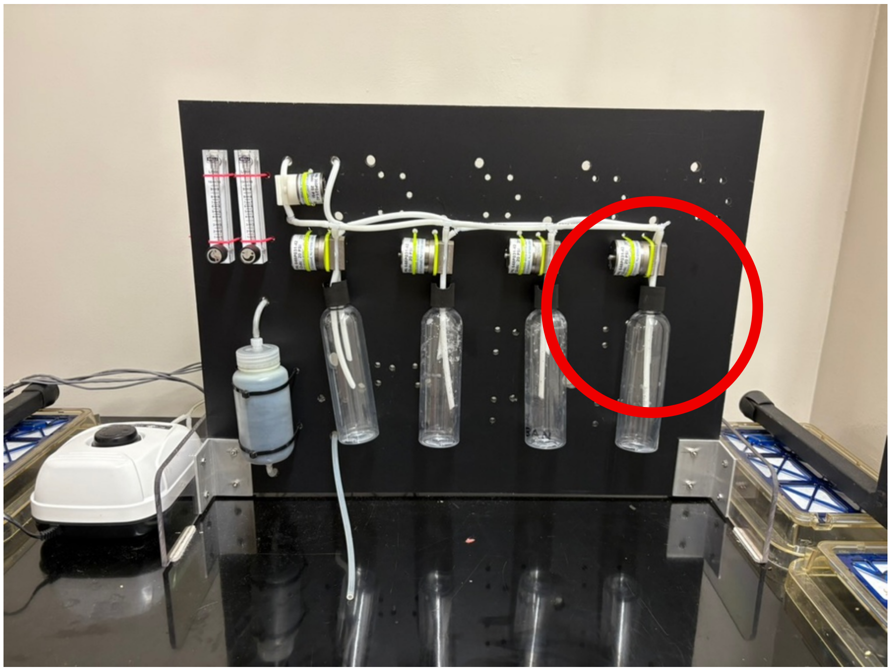

V) Insert a free section of tubing into the *odorant bottle* and then into the corresponding *odorant valve* to form the final *odorized air feed branch*. Use a wye connector to combine the *odorized air feed branch* into the *stimulus air feed line*.

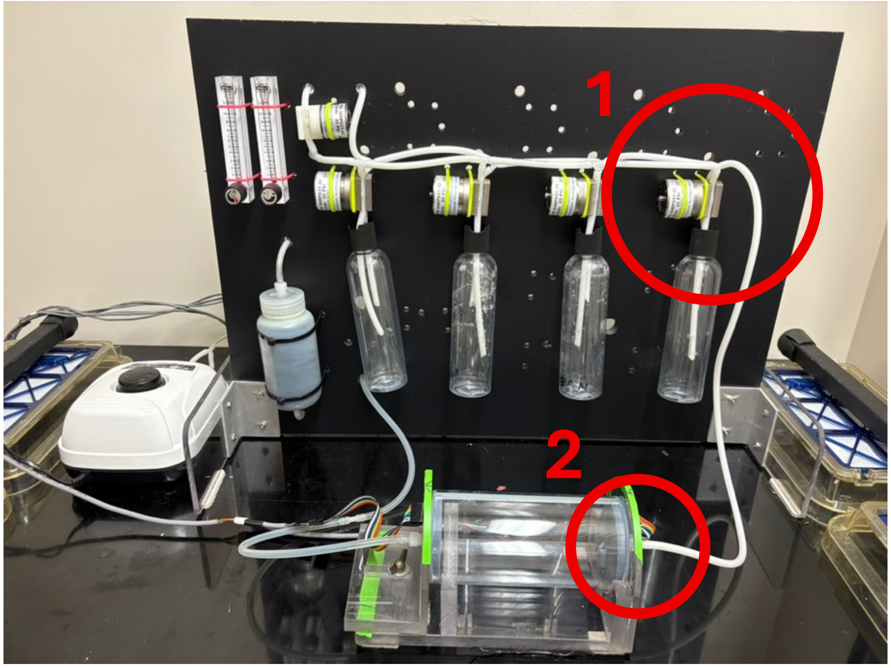

W) Connect the last wye connector on the *stimulus air feed line* (**1**) to the *primary side* (**2**) of the *testing chamber* using a long section of tubing.

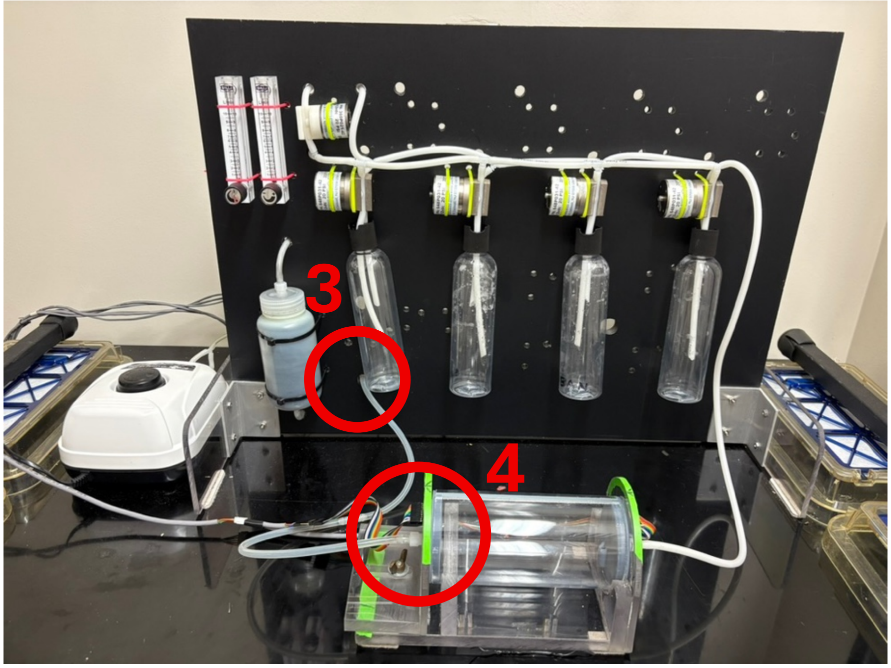

X) Connect the *secondary air feed line* (**3**; **Step H**) to the *secondary side* (**4**) of the *testing chamber*.

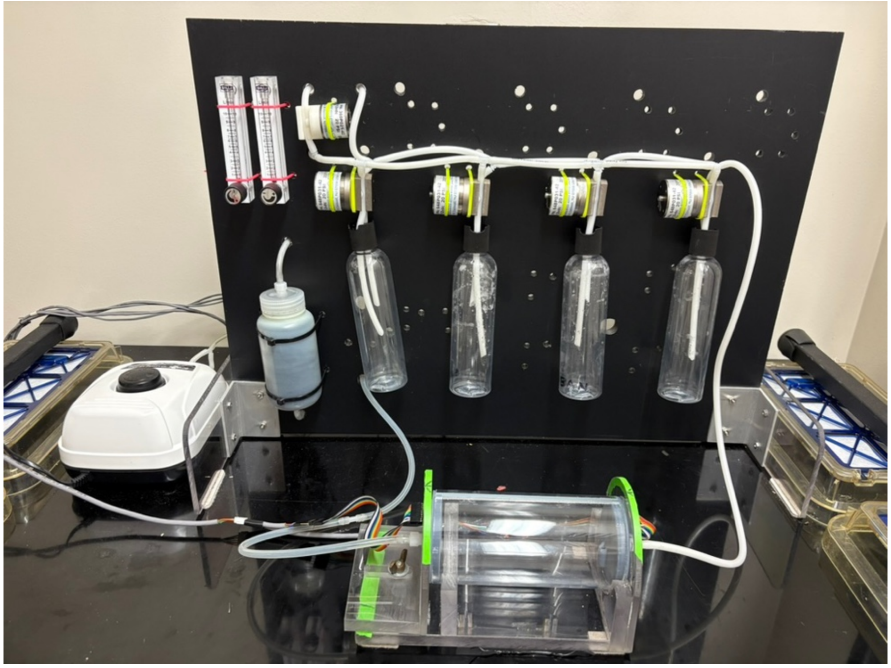

Y) Connect the wiring for the *clean air valve* and the *odorized air valves* to their respective triggers. If using VOC sensors, install and connect them to the Arduino (or other single-board computer).

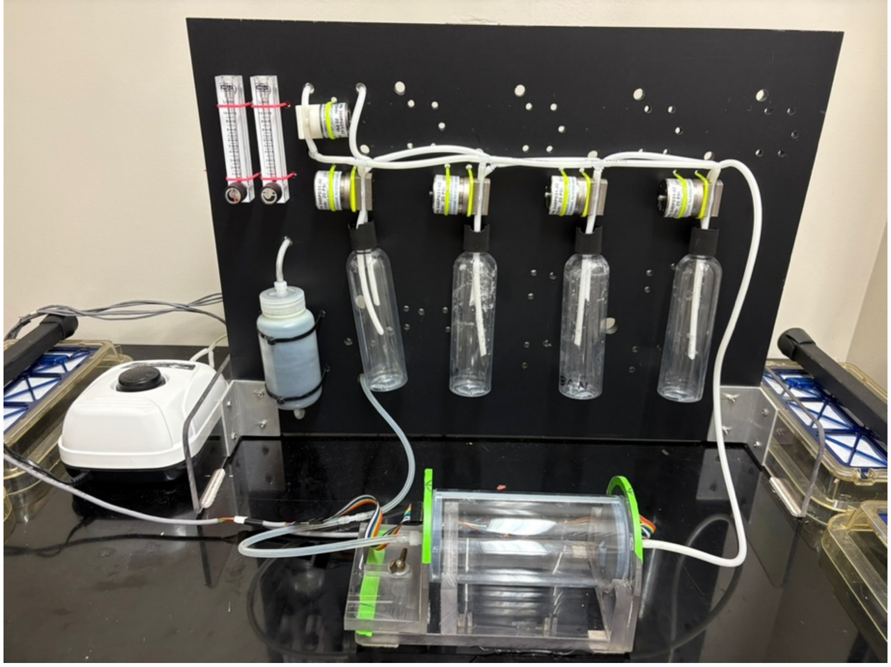

Z) Fill the *carbon filter* with the selected filter media (if not yet done) and fully open both *rotameter valves*. Overall airflow should be adjusted at the *air pump* while *rotameter valves* should be used to fine-tune airflow for *primary* and *secondary air lines*; this will allow the air pump to run at a lower speed to reduce wear and noise.

AA) The following figure provides a basic outline of airflow through the ODORS to aid in understanding how each component is connected. The following figures provide a guide for attaching each component and assembling the board. Information on specific components (e.g., brand; function) is detailed in the associated manuscript.

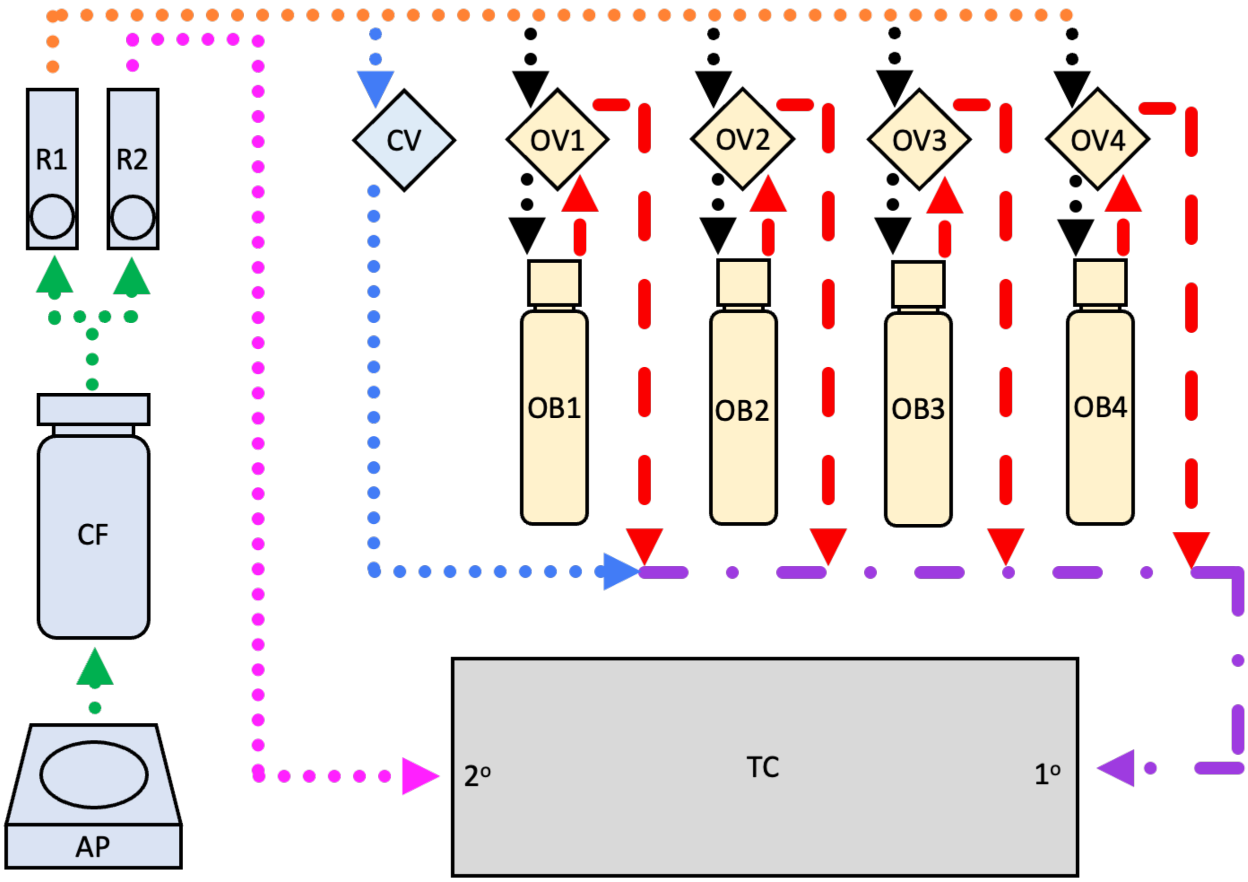

Simplified air flow diagram. The *main air supply* (green) from the *air pump* (AP) passes through the *carbon filter* (CF) before being entering the *primary* and *secondary rotameters* (R1, R2). The *primary air supply line* (orange) exits from the top of the *primary rotameter*. The *primary air supply line* branches into the *clean air feed line* (blue) and passes through the *clean air valve* (CV). Additional *odorant bottle supply branches* (black) pass through a single *odorant valve* (OV#) to supply air to a single *odorant bottle* (OB#). Each *odorant valve/bottle* pair uses a separate *odorant bottle supply branch*. An *odorized air feed branch* (red) passes odorized air from each *odorant bottle* through the respective *odorant valve*. The first *odorized air feed branch* is joined with the *clean air feed branch* to form the start of the *stimulus air feed line* (purple). Each subsequent *odorized air feed branch* is then connected directly to the *stimulus air feed line*. The *stimulus air feed line* is connected to the primary (1˚) end of the *testing chamber* (TC) to provide stimulus air. A *secondary air feed line* (pink) is connected to the secondary (2˚) end of the *testing chamber*.
