# Supplementary material for: The Odor Delivery Optimization Research System (ODORS): An Open-Source Olfactometer for Behavioral Assessments in Tethered and Untethered Rodents
Source: eNeuro. 2025 Dec 17;12(12):ENEURO.0161-25.2025. doi: 10.1523/ENEURO.0161-25.2025 (PMC12757508; doi:10.1523/ENEURO.0161-25.2025)
Supplement: Data 1 — Download Data 1, ZIP file. [file eneuro-12-ENEURO.0161-25.2025-s001.zip › ODORS-main/Olfactometer-Bill_of_Materials.pdf]

Olfactometer Bill of Materials

Assembly	Component	Product and Brand	Part Number	Description	Manufacturer	Supplier*
Air Pump	Air pump	EcoPlus Eco Air 4	#HGC728355	Aquarium air pump; 253 GPH; adjustable flow rate; 4-outlet; low noise	Hawthorne Gardening Company, Vancouver, WA	Local retailer
Air Line	Air line	C-Flex tubing	#06424-67	Silicone tubing; 1/8” (3.5mm) ID; 1/4" (7mm) OD	Cole-Parmer, Vernon Hills, IL	VWR
Olfactory Board	Air filter – Outer	Nalgene	#2104-0016	500mL HDPE bottle; wide-mouth	Nalge Nunc International, Rochester, NY	University stores
	Air filter – Media	Seachem Matrix Carbon		Carbon; spherical beads	Seachem Laboratories, Madison, GA	Local retailer
	AIr rotameter (x2)	LZQ-7	#LZQ-7	Rotameter; 1-10L/min	Yuyao Shunhuan Flowmeter Co., Yuyao, China	Amazon
	Odorant valves (x4)	NResearch	#648P03-42	Solenoid pinch valve; 12VDC; 2-outlet; normally-closed; 1/4" OD	NResearch Inc., West Caldwell, NJ	NResearch
	Clean air valve (x1)	NResearch	#360P021-42	Solenoid pinch valve; 12VDC; 1-outlet; normally-open; 1/4" OD	NResearch Inc.	NResearch
	Odorant bottles – Bottle (x4)	N/A	N/A	250mL narrow-top; polyethylene terephthalate (PET); clear	N/A	Dr. RE Brown
	Odorant bottles – Caps (x4)	N/A	N/A	Disc cap; 24-410 size; polypropylene; black; disc	N/A	Dr. RE Brown

				section removed and second 1/4" hole drilled opposite to existing opening		
	Air line connectors - Wye	McMaster-Carr “Super-Flow”	#2808K127	Barbed connectors; Wye; polyethylene; 1/8” tube	McMaster-Carr, Elmhurst, IL	Master-Carr
	Air line connectors - Tee	McMaster-Carr “Super-Flow”	#2808K166	Barbed connectors; Tee; polyethylene; 1/8” tube	McMaster-Carr	Master-Carr
	Air line connectors – 90-degree Elbow	McMaster-Carr “Super-Flow”	#2808K115	Barbed connectors; 90-degree elbow; polyethylene; 1/8” tube	McMaster-Carr	Master-Carr
	Air line connectors – Barbed/threaded (air filter; testing chamber)	McMaster-Carr “Super-Flow”	#2808K22	Barbed/threaded connectors; polyethylene; 1/8” tube x male 1/8” NPT	McMaster-Carr	Master-Carr
	Air line connectors – Barbed/threaded (rotameter)	McMaster-Carr	#622N104	Barbed/threaded connectors; nickel-plated brass; 4mm tube x 1/16” BSPT	McMaster-Carr	Master-Carr
	Mounting board	N/A	N/A	Low-density polyethylene (LDPE); 3’ x 2’; mounted upright using acrylic feet	N/A	University workshop
Testing Chamber	Testing chamber	N/A	N/A	Transparent acrylic tube; 2-3/4” ID x 6” long; 1/8” slot cut lengthwise from end to end	N/A	University workshop

	Testing chamber holder	N/A	N/A	Transparent acrylic blocks; see manuscript for figures and description		University workshop
	End caps	3D printed	N/A	One primary end cap with odour tube, one secondary perforated end cap; see GitHub for .stl files	DIY	DIY
Arduino	Microcontroller	Arduino	Uno	Arduino Uno; USB cable connection to computer; 9VDC power supply for constant power (if desired)	Arduino, Monza, Italy	Digikey
	VOC sensors (x2)	DFRobot	CCS811 Air Quality Sensor	CO2 and tVOC air quality sensors; 4-pin header; address selection pads soldered on one board for secondary address (0x5A and 0x5B addresses); WAKE pin shorted to GND for constant operation	DFRobot, Shanghai, China	Digikey
	Arduino to sensor cable	Alpha Wire	1175C	5-conductor cable (5VDC, ground, SDA, and SCL); 6' long; connectors attached for Arduino and VOC sensors	Alpha Wire, Elizabeth, NJ	Digikey
	Arduino to interface box cable	Alpha Wire	1175C	5-conductor cable (1 conductor per odorant valve + ground); 3' long; connectors attached for Arduino	Alpha Wire	Digikey

Computer Interface/Valve Power Supply	Power supply	TDK-Lambda	LS100-12	120VAC to 12VDC power supply	TDK-Lambda Americas Inc., Neptune, NJ	Digikey
	Solid-state relays (x5)	N/A	N/A	Opto-coupled solid-state relays; 1 per valve	N/A	University workshop (Digikey)
	Screw terminals	N/A	N/A	Used for connecting interface box to Arduino and valves	N/A	University workshop (Digikey)

Note: N/A is used for generic components where the specific manufacturer and/or brand is not available and not critical to the function of the apparatus. Several components were obtained locally, either through the University workshop, University stores, or a local retailer, although these may be available from other sources (including online). For 3D models of end caps, Arduino code, and the Python script, visit: https://github.com/FilipKosel/ODORS
